# Supplementary material for: Comparative genomic analysis reveals metabolic flexibility of Woesearchaeota
Source: Nat Commun. 2021 Sep 6;12:5281. doi: 10.1038/s41467-021-25565-9 (PMC8421398; doi:10.1038/s41467-021-25565-9)
Supplement: Supplementary file 1 — Supplementary Information [file 41467_2021_25565_MOESM1_ESM.pdf]

Supplementary Information

Comparative genomic analysis reveals metabolic flexibility of Woesearchaeota

Wen-Cong Huang[1]†, Yang Liu[1]†, Xinxu Zhang[1], Cui-Jing Zhang[1], Dayu Zou[1,2], Shiling Zheng[3], Wei Xu[4], Zhuhua Luo[4,5], Fanghua Liu[3,6], Meng Li[1*]

[1] Shenzhen Key Laboratory of Marine Microbiome Engineering, Institute for Advanced Study, Shenzhen University, Shenzhen 51800, China

[2] Department of Ocean Science, The Hong Kong University of Science and Technology, Hong Kong SAR 999077, China

[3] Key Laboratory of Coastal Biology and Biological Resources Utilization, CAS Key Laboratory of Coastal Environmental Processes and Ecological Remediation, Yantai Institute of Coastal Zone Research, Chinese Academy of Sciences, Yantai 264003, China

[4] Key Laboratory of Marine Biogenetic Resources, Third Institute of Oceanography, Ministry of Natural Resources, Xiamen 361005, China

[5] School of Marine Sciences, Nanjing University of Information Science & Technology, Nanjing 210044, China

[6] National-Regional Joint Engineering Research Center for Soil Pollution Control and Remediation in South China, Guangdong Key Laboratory of Integrated Agro-environmental Pollution Control and Management, Institute of Eco-environmental and Soil Sciences, Guangdong Academy of Sciences, Guangzhou 510650, PR China

***CORRESPONDENCE:** Meng Li, Room 360, Administration Building, Institute for Advanced Study, Shenzhen University, Shenzhen, China; E-mail: limeng848@szu.edu.cn; Tel: +86-755-26979250.

† These authors contributed equally.

Supplementary Note 1

Subgroup definition from the reference paper was consequently updated[1], as follows: (1) Woese-5 were found to encompass sequences belonging to Pacearchaeota; (2) Woese-22 were removed as the 16S rRNA gene sequences did not cluster within Woesearchaeota; and (3) Woese-14 and Woese-21 were further split into two sister groups (Supplementary Fig. 5).

Supplementary Note 2

Subgroups in different trees.

In the phylogenetic analyses, the tree based on the 50% top-ranked orthologs (UFBOOT: 91% and SH-aLRT: 94%, on average) is more statistically supported than the tree based on 15 ribosomal proteins (rps) (UFBOOT: 88% and SH-aLRT: 89%, on average). They have a Robinson-Foulds distance of 96.

Subgroup A, B, C, G and J are monophyletic in the orthologs tree and 15rps tree (Supplementary Fig. 44). The rest subgroups D, E, F, H and I are paraphyletic in the 15rps tree and formed some contradicting groupings. However, the grouping is not statistically well-supported (UFBOOT < 95%; SH-aLRT < 80%). For example, in 15 rp tree, MP5_1_678 and YT1_142 of subgroup H form sister to the subgroup C with weak support (56% UFBOOT and 71% SH-aLRT) and, the clade formed by T1Sed10_208R1, CG10_big_fil_rev_8_21_14_0_10_34_8 and subgroup G also has poor support (73.4% UFBOOT and 68% SH-aLRT). Similarly, the sisterhood of UBA10107, YT2_062, UBA10192, CG10_big_fil_rev_8_21_14_0_10_44_13 and MP5_7_64 to subgroup H and J in the 15 rp tree is not strongly supported (96% UFBOOT and 77% SH-aLRT). Considering that the trimmed alignments of the 15 ribosomal proteins were relatively short (2734 amino acids) and have only 2488

parsimony-informative sites, they likely harbor insufficient information to resolve groupings in the Woesearchaeota as indicated by the low support.

The sequence clusters based on 16S rRNA gene phylogenetic analysis agree well with the tree based on the 50% top-ranked orthologs. Taking advantage of 16S rRNA gene sequence recovered from genomes, several previous defined sequence clusters are able to link with genome-based subgroups. Woese-3, Woese-4, Woese-21a, Woese-24, Woese-14a and Woese-14b correspond to subgroup A, C, J, I, H and E respectively. In addition, subgroup G could be linked with a monophyletic clade in 16S rRNA gene tree including Woese-8, Woese-10, Woese-9, Woese-6, Woese-18 and Woese-20, indicating that a large part of subgroup G diversity remains unexplored. Subgroup D was paraphyletic in the 16S rRNA gene tree, whose sequence representatives were found in Woese-21b and Woese-14a. However, it should be noted that more 16S rRNA gene sequences retrieved from genomes are required to accurately describe their relationship.

Supplementary Tables

Supplementary Table 1. Mantel test between Euclidean distance matrix of available physiochemical parameters in metadata of EMP datasets and unweighted UniFrac matrix of corresponding Woesearchaeota community. The test was done using "mantel" function in vegan package in R (3.6.0). Pearson correlation was used and significance level was obtained from 999 permutations. Number of libraries indicates the number of libraries with parameters available for the correlation test. Mantel statistic r, ranging for -1 (negative) to 0 (no effect) to 1 (positive), measures the strength of the relationship.

Parameter	Mantel statistic (r)	Significance	Number of libraries
temperature (°C)	0.167461	0.001	726
pH	0.1245451	0.001	593
salinity (psu)	0.2956271	0.001	167
oxygen (mg/L)	0.2113615	0.021	37
phosphate (μmol/L)	0.3474911	0.001	226
ammonium (μumol/L)	0.2128725	0.001	63
nitrate (μmol/L)	0.4036696	0.001	457
sulfate (μmol/L)	0.011915	0.356	152

Supplementary Table 2. The linkage of 16S rRNA gene sequence clusters and genome-based subgroups.

Genome-based subgroups	16S rRNA gene sequence clusters	Monophyly of gene sequence clusters
Subgroup A	Woese-3	Yes
Subgroup C	Woese-4	Yes
Subgroup D	Woese-21b, Woese-23b	No
Subgroup E	Woese-14b	Yes
Subgroup G	Woese-8, Woese-10, Woese-9, Woese-6, Woese-18, Woese-20	Yes
Subgroup H	Woese-14a	Yes
Subgroup I	Woese-24	Yes
Subgroup J	Woese-21a	Yes

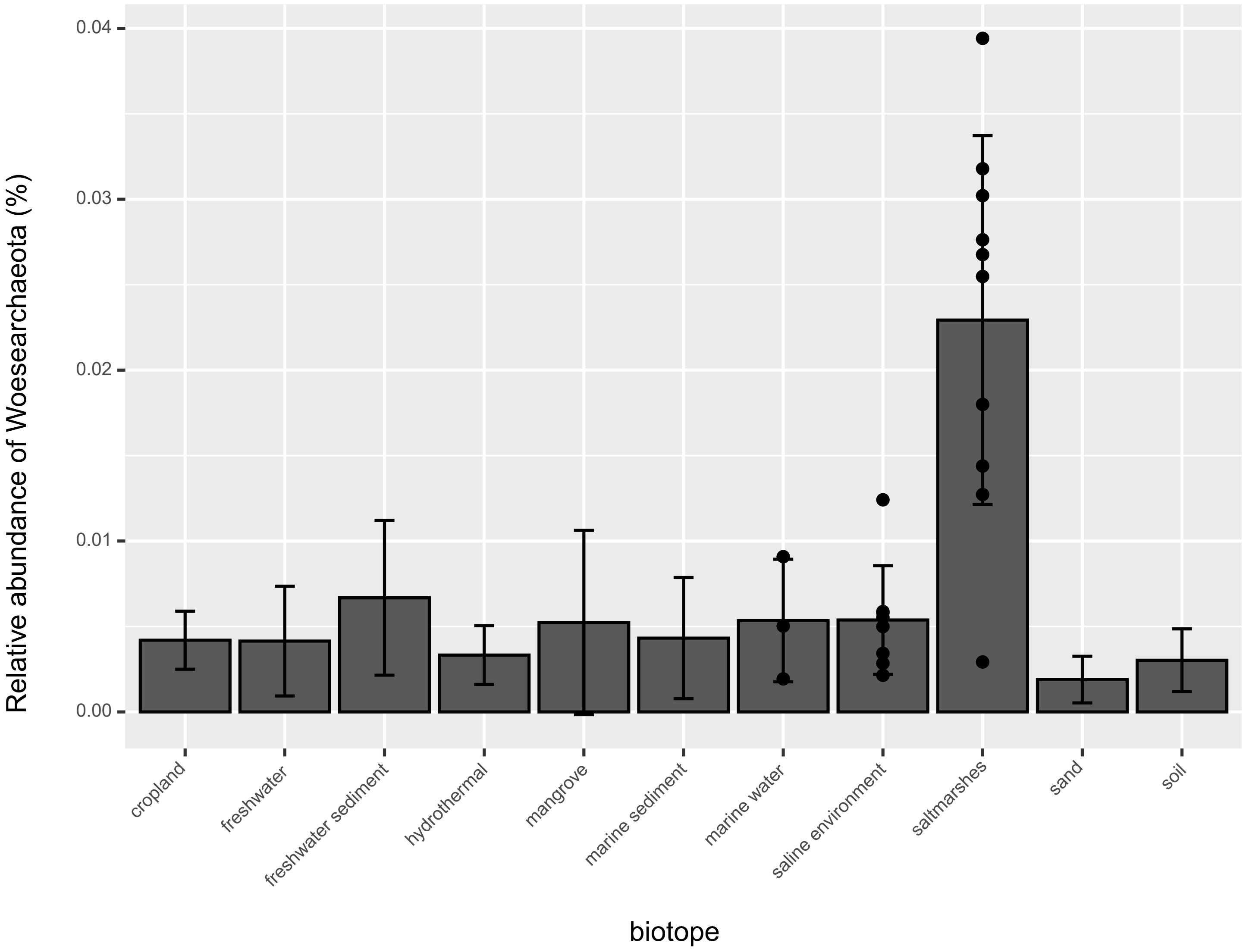

Supplementary Fig. 1: The average relative abundance of Woesearchaeota based on 2163 16S rRNA gene amplicon dataset. Data are presented as mean + / - SD. The number of replicates used for different biotopes are as follows: freshwater: 629, cropland: 590, freshwater sediment: 365, marine sediment: 293, sand: 174, mangrove: 46, soil: 30, hydrothermal: 15, saltmarshes: 10, saline environment: 8, marine water: 3. Source data are provided as a Source Data file.

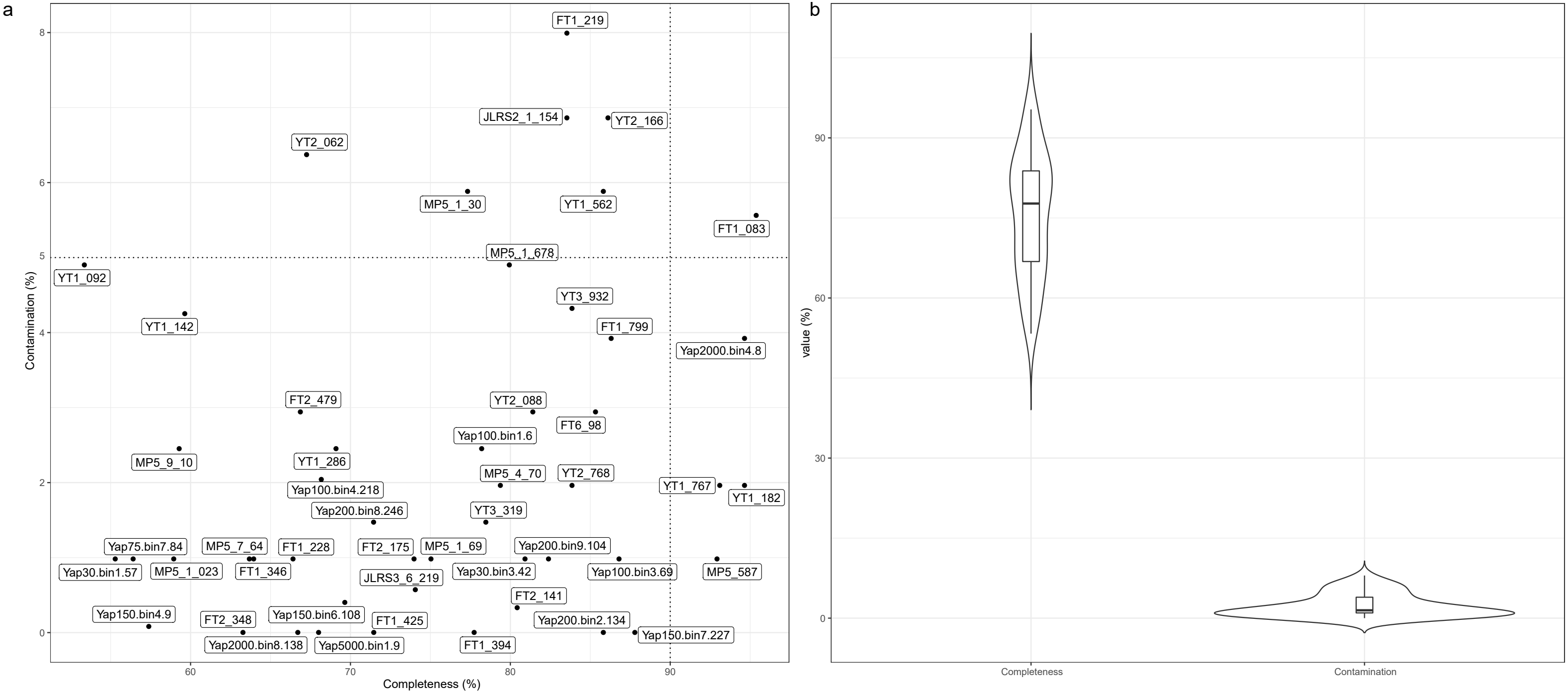

Supplementary Fig. 2: Estimated quality statistics of 49 Woesearchaeota metagenome-assembled genomes (MAGs). Completeness and contamination were estimated with CheckM (v 1.0.12) as described in the method section and raw values could be found in Supplementary Data 2. (**a**) Plot of estimated contamination versus estimated completeness. Each point denotes a MAG and its name is labeled alongside. Dashed line shows the completeness threshold of 90% and contamination threshold of 5%. (**b**) Violin plot and box plot of estimated genome quality metrics. The shape of violin plot represents a kernel density estimation to show probabilistic distribution of the data (n=49). Median of the estimated completeness (77.7 %) and contamination (1.47 %) are shown as the thick black bar. The upper and lower hinges of the box plot indicate the first and third quartile respectively. The upper/lower whiskers stretch out of the hinge to the largest/smallest value less than 1.5 * interquartile range. Source data are provided as a Source Data file.

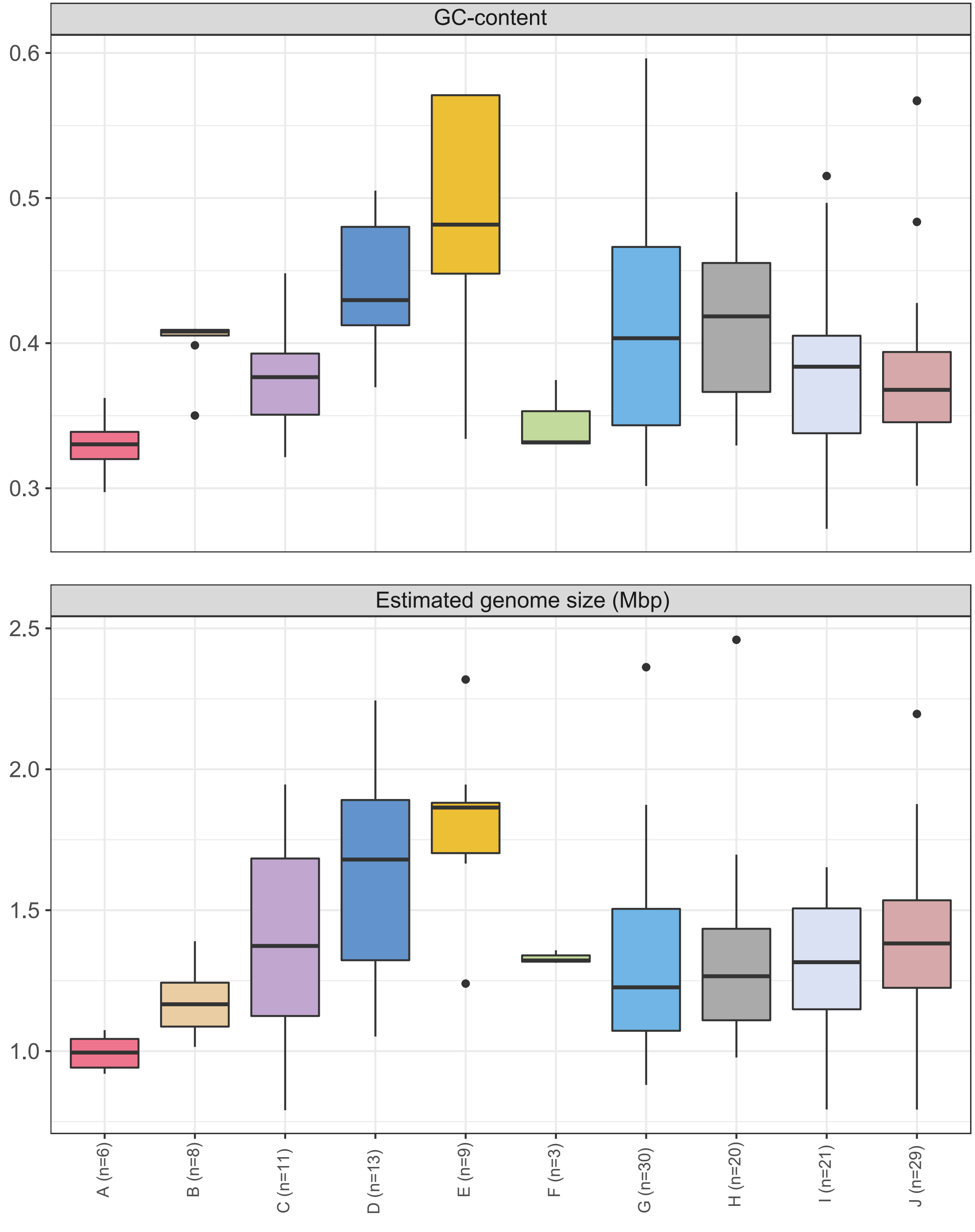

Supplementary Fig. 3: Box plot of general genomic features (GC-content and estimated genome size) of Woesearchaeota MAGs (n=150) of different subgroups. The number of MAGs in each subgroup is shown in parenthesis. GC-content was calculated by CheckM (v 1.0.12). Estimated genome size is calculated by dividing the MAG size by the estimated completeness and then multiplying 100. Thick black bar shows the median value. The upper and lower hinges of the boxplot indicate the first and third quartile respectively. The upper/lower whiskers stretch out of the hinge to the largest/smallest value less than the 1.5 * interquartile range. Colors were used to distinguish different subgroups according to the Fig. 2. The black points correspond to the values great than the 1.5 * interquartile range. Source data are provided as a Source Data file.

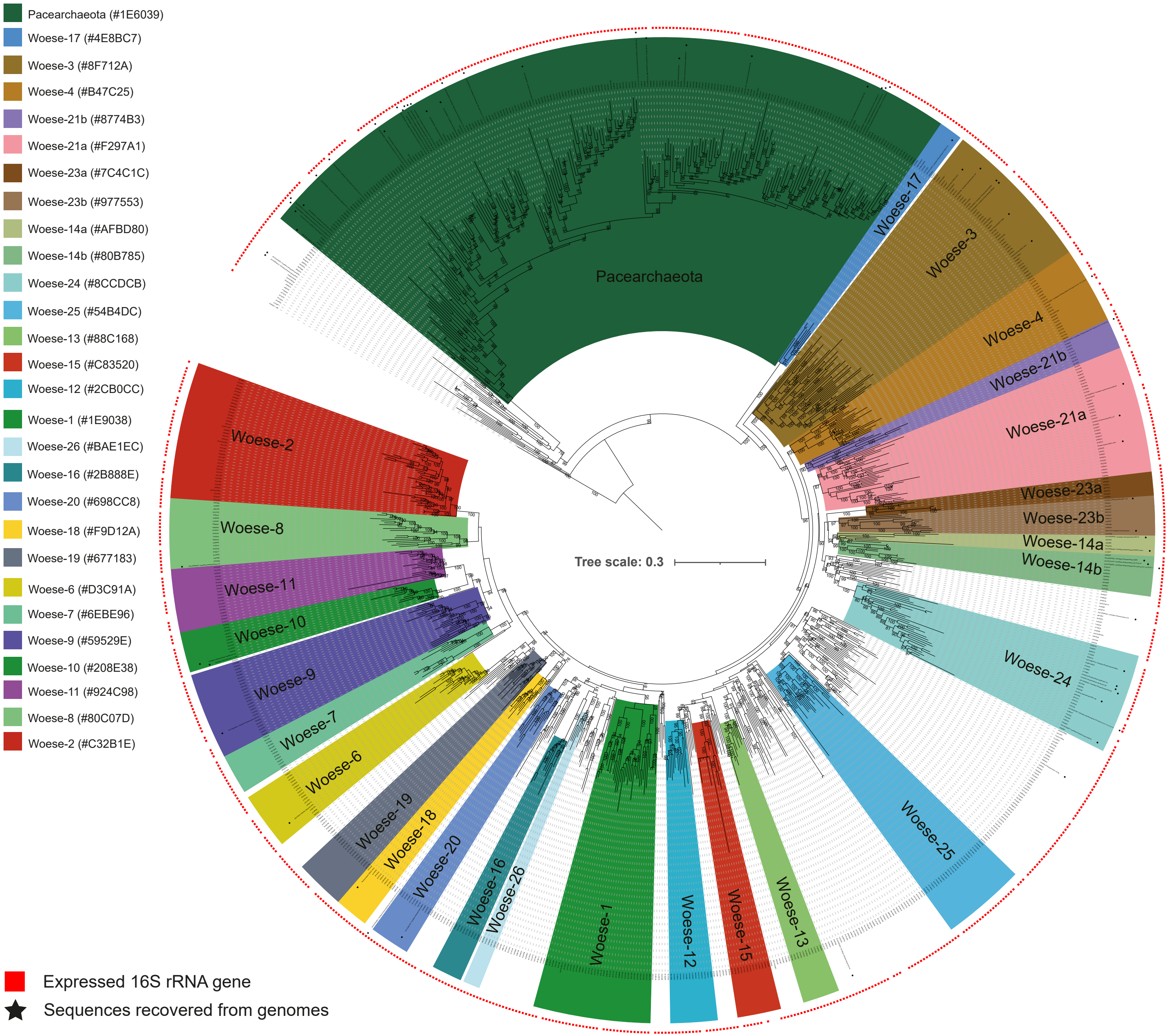

Supplementary Fig. 4: Phylogenetic analysis based on 16S rRNA gene sequences using IQ-Tree (v 1.6.8) based on the TIM+F+G model. The tree includes 891 16S rRNA gene sequences (35 from Woesearchaeota MAGs, 28 from Pacearchaeota MAGs, and three from unclassified MAGs, 823 from SILVA SSU 132 database or Liu et al.[1]) and was rooted using two sequences from Nanoarchaeota. The ultrafast bootstrap values are shown at the nodes. The hex code for the color of each sequence cluster was shown at the legend. Stars denote 16S rRNA gene sequences retrieved from genome and red rectangles indicate a shared sequence identity (>97%) with sequences in an expressed 16S rRNA gene dataset[2]. Scale bar shows the average nucleotide substitutions per site. Source data are provided as a Source Data file.

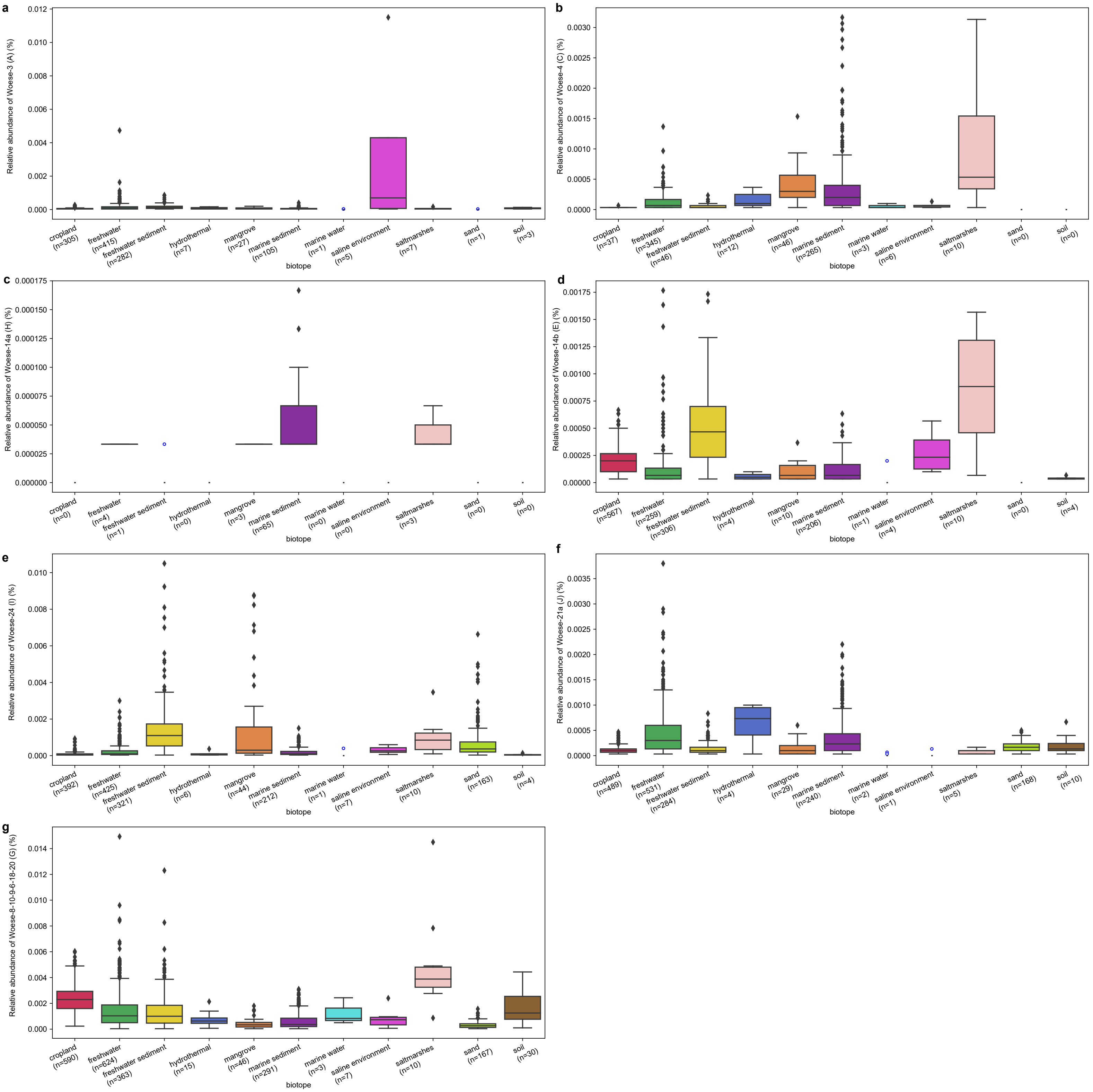

Supplementary Fig. 5: Distribution of Woesearchaeota subgroups in the studied biotopes. a-g, Distribution of Woese-3 that is linked with subgroup A (**a**), Woese-4 that is linked with subgroup C (**b**), Woese-14a that is linked with subgroup H (**c**), Woese-14b that is linked with subgroup E (**d**), Woese-24 that is linked with subgroup I (**e**), Woese-21a that is linked with subgroup J (**f**) and the aggregate of Woese-8, Woese-10, Woese-9, Woese-6, Woese-18 and Woese-20 that are linked with subgroup G (**g**) in the 2163 investigated libraries. Colors were used to distinguish different biotopes according to the Fig. 1. Thick black bar shows the median value. The upper and lower hinges of the boxplot indicate the first and third quartile respectively. The upper/lower whiskers stretch out of the hinge to the largest/smallest value less than 1.5 * interquartile range. The black points correspond to the values great than the 1.5 * interquartile range. Data less than 3 are shown as hollow blue points. The number of datasets for each biotope used in each panel are as follows: cropland: (a: 305, b: 37, c: 0, d: 567, e: 392, f: 489, g: 590), freshwater: (a: 415, b: 345, c:4, d: 259, e: 425, f: 531, g: 624), freshwater sediment: (a: 282, b: 46, c:1, d: 306, e: 321, f: 284, g: 363), hydrothermal: (a: 7, b: 12, c:0, d: 4, e: 6, f: 4, g: 15), mangrove: (a: 27, b: 46, c:3, d: 10, e: 44, f: 29, g: 46), marine sediment: (a: 105, b: 265, c: 65, d: 206, e: 212, f: 240, g: 291), marine water: (a: 1, b: 3, c: 0, d: 1, e: 1, f: 2, g: 3), saline environment (a: 5, b: 6, c: 0, d: 4, e: 7, f: 1, g: 7), saltmarshes (a: 7, b: 10, c: 3, d: 10, e: 10, f: 5, g: 10), sand (a: 1, b: 0, c: 0, d: 0, e: 163, f: 168, g: 167), and soil (a: 3, b: 0, c: 0, d: 4, e: 4, f: 10, g: 30). Source data are provided as a Source Data file.

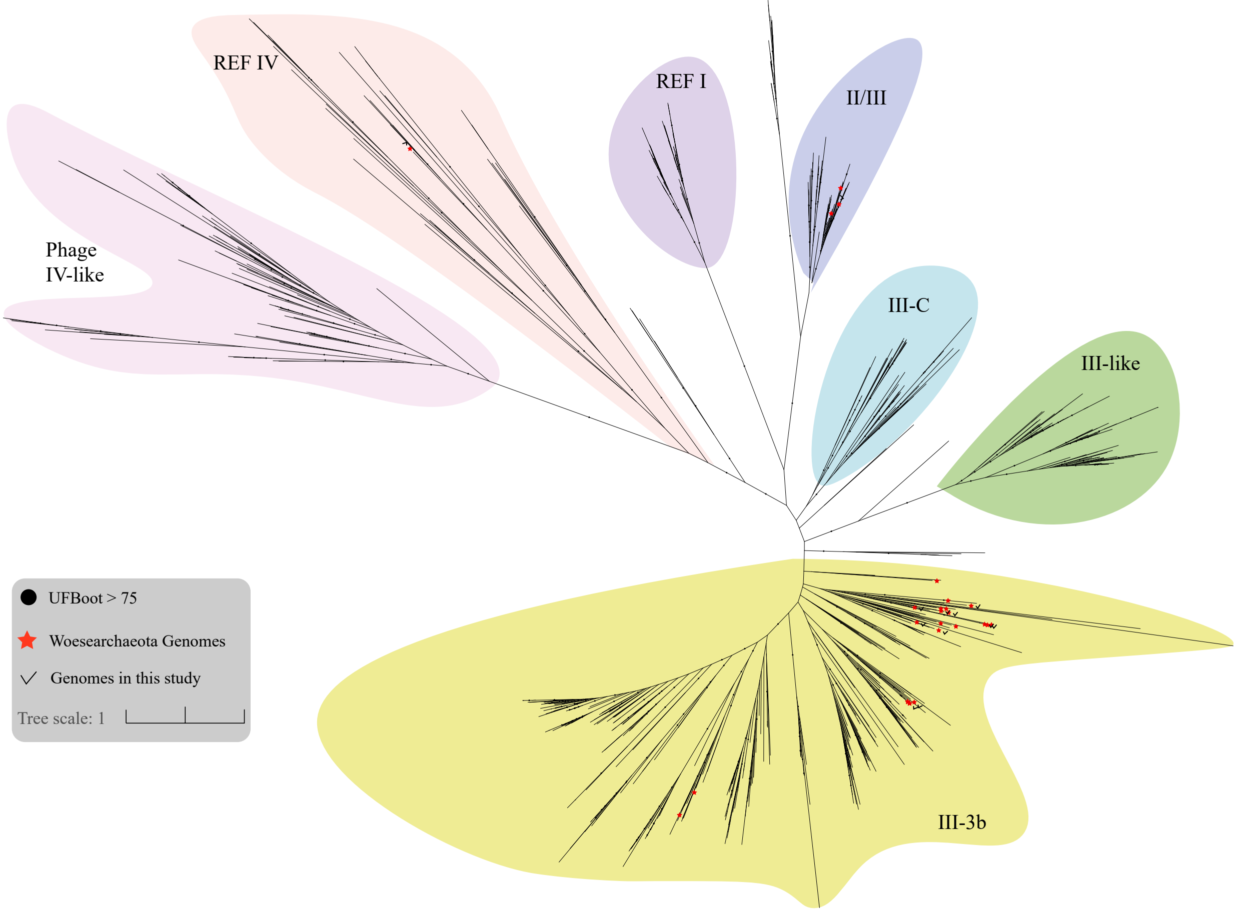

Supplementary Fig. 6: Phylogenetic analysis of ribulose 1,5-bisphosphate carboxylase large subunit (*rbcL)* based on LG+G model using IQ-Tree. The tree is unrooted and includes an alignment of 776 sequences of 163 sites. Scale bar shows the average number of substitutions per site. Source data are provided as a Source Data file and raw data are also available via figshare (See Data availability for more details).

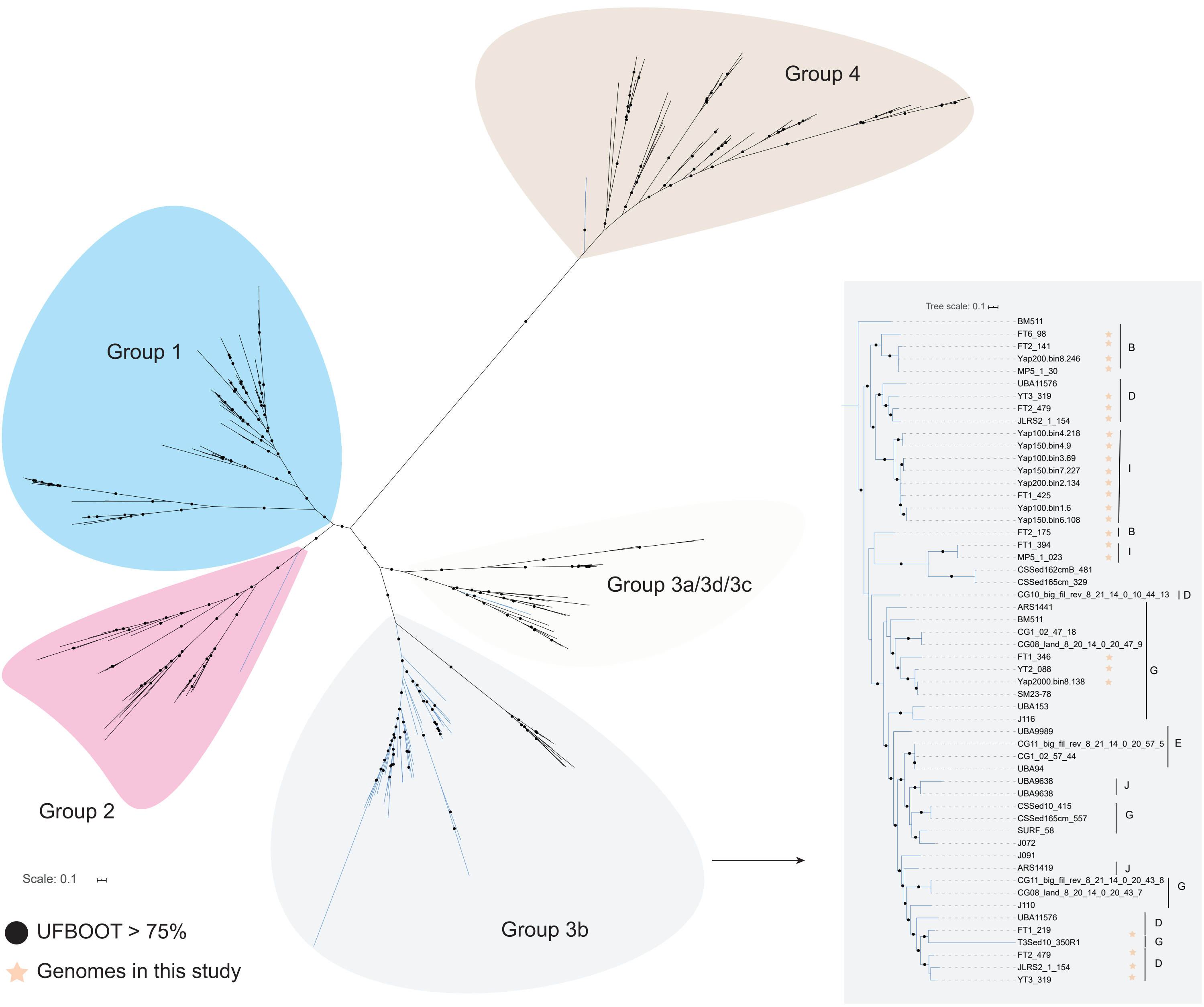

Supplementary Fig. 7: Phylogenetic analysis of [NiFe] hydrogenase based on LG+G model. The tree is unrooted and includes an alignment of 277 sequences of 169 sites. Scale bar shows the average number of substitutions per site. Inset shows phylogenetic clades of [NiFe] hydrogenase group 3b of Woesarchaeota. Source data are provided as a Source Data file and raw data are also available via figshare (See Data availability for more details).

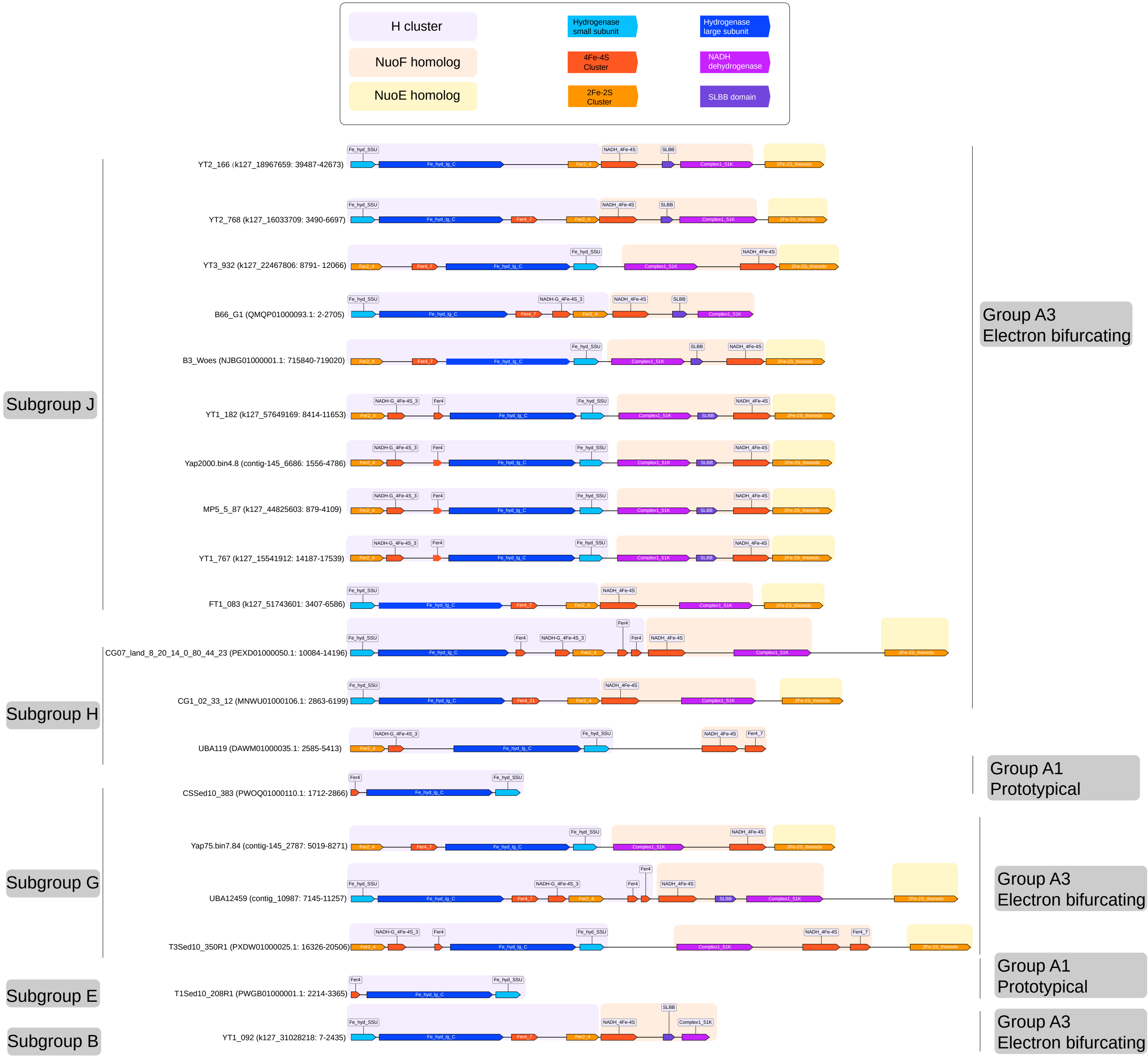

Supplementary Fig. 8: Genomic regions encoding [FeFe] hydrogenase complex in Woesearchaeota MAGs. All genes are drawn to scale. Detailed location of [FeFe] hydrogenase is shown in the parenthesis by the format (contig name: starting bases - ending bases). Most [FeFe] hydrogenase in Woesearchaeota is trimeric, containing a H cluster, NuoF homologue and NuoE homologue. [FeFe] hydrogenase large subunit and small subunit, consisting of the catalytic domains, are respectively marked in dark and light blue. Dark orange and light orange correspond to 4Fe-4S cluster and 2Fe-2S cluster respectively. NADH dehydrogenase domain and soluble ligand binding domain are colored in respectively in dark and light purple. Domains were annotated by Pfam (v 32.0) by InterProScan (v 5.38-76.0, client version). The corresponding domain names and Pfam accessions are shown as below: Fe_hyd_SSU: PF02256, Fe_hyd_lg_C: PF02906, Fer2_4: PF13510, NADH-G_4Fe-4S_3: PF00037, Fer4: PF02906, Complex1_51K: PF01512, SLBB: PF10531, NADH_4Fe-4S: PF10589, 2Fe-2S_thioredx: PF01257, Fer4_7: PF12838, Fer4_21: PF14697.

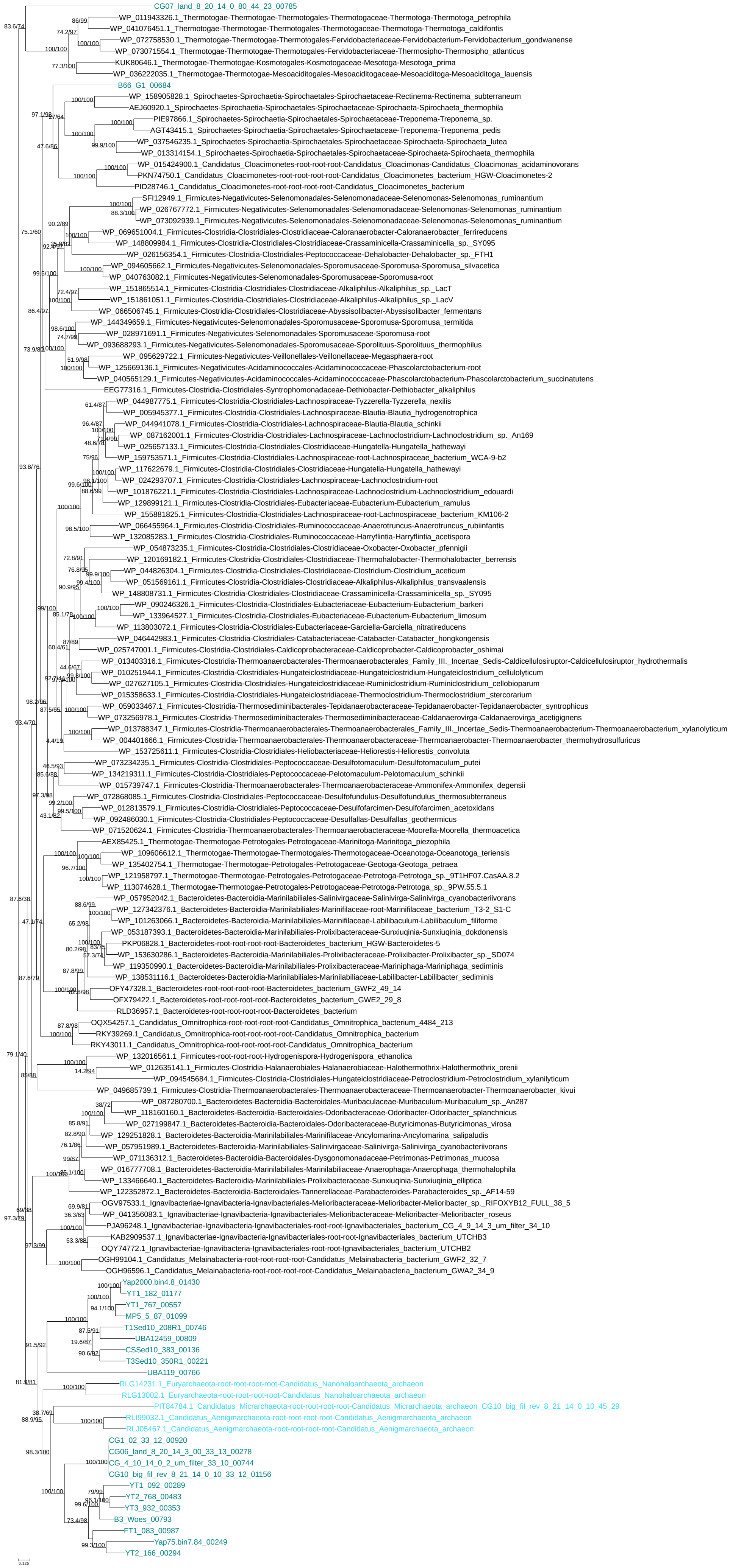

Supplementary Fig. 9: Phylogenetic analysis of catalytic subunit of [FeFe] hydrogenase in Woesearchaeota. The tree was unrooted and inferred using IQ-Tree LG+G+C20 model on an alignment of 216 taxa and 598 sites. UFBOOT (left) and SH-aLRT (right) support values are shown along the branches. Bacteria, Archaea and Woesearchaeota are colored in black, cyan and green respectively. Scale bar shows the number of average substitutions per site. Source data are provided as a Source Data file and raw data are also available via figshare (See Data availability for more details).

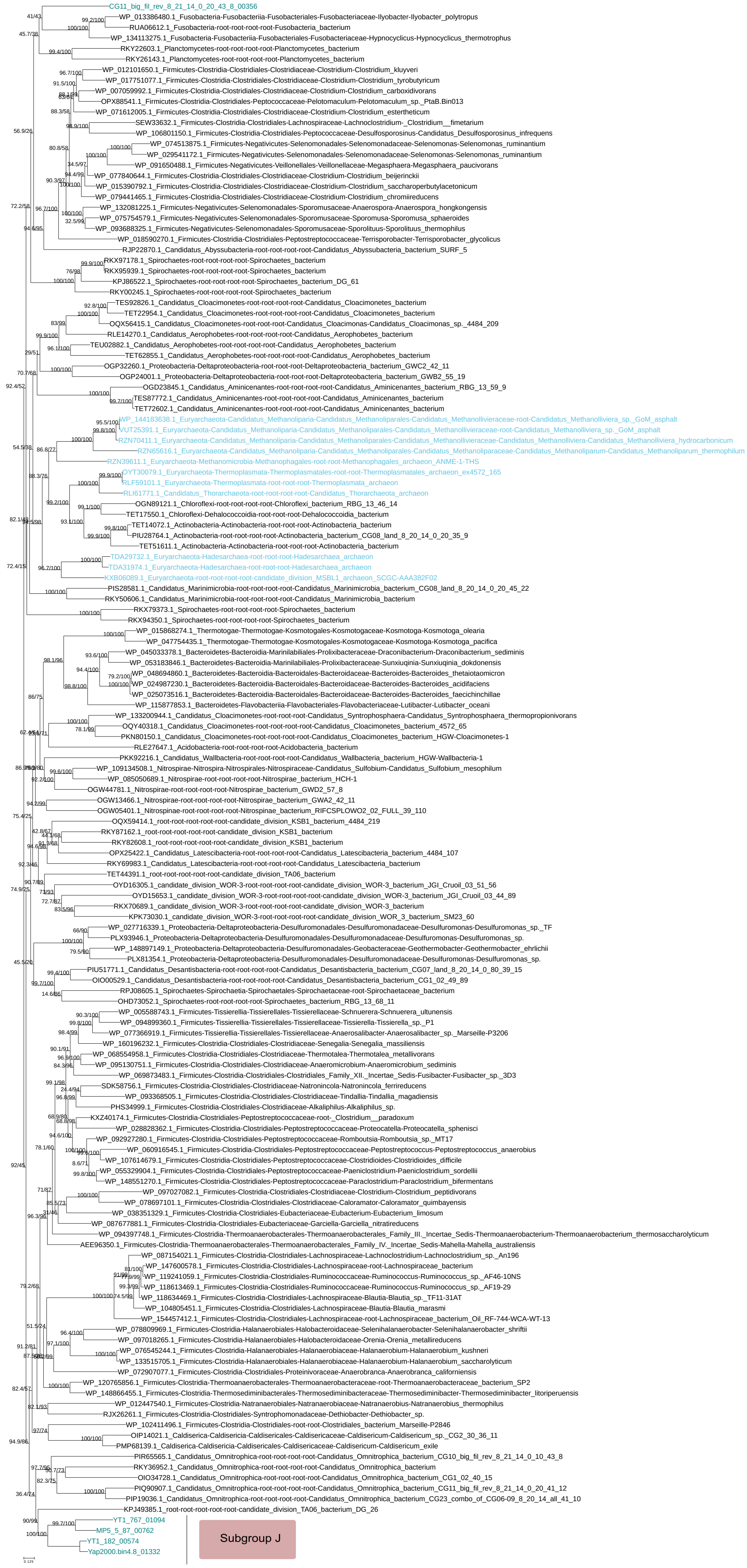

Supplementary Fig. 10: Phylogenetic analysis of *rnfC* gene (*Rhodobacter* nitrogen fixation complex subunit C). The tree was unrooted and inferred using IQ-Tree LG+C20+G model on an alignment of 147 taxa and 440 sites. UFBOOT (left) and SH-aLRT (right) support values are shown along the branches. Bacteria, Archaea, and Woesearchaeota are colored in black, cyan, and green, respectively. Scale bar shows the number of average substitutions per site. Source data are provided as a Source Data file and raw data are also available via figshare (See Data availability for more details).

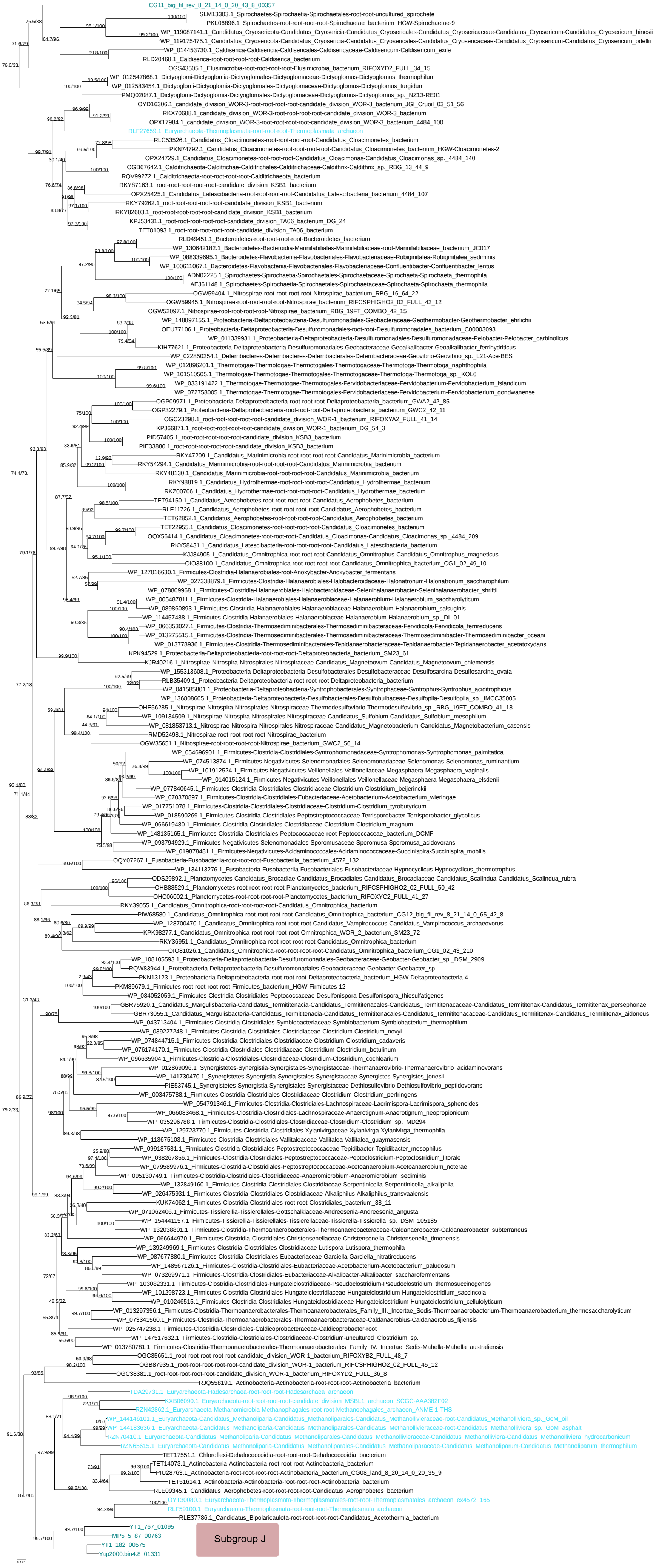

Supplementary Fig. 11: Phylogenetic analysis of *rnfD* gene (*Rhodobacter* nitrogen fixation complex subunit D). The tree was unrooted and inferred using IQ-Tree LG+C20+G model on an alignment of 173 taxa and 339 sites. UFBOOT (left) and SH-aLRT (right) support values are shown along the branches. Bacteria, Archaea and Woesearchaeota are colored in black, cyan, and green, respectively. Scale bar shows the number of average substitutions per site. Source data are provided as a Source Data file and raw data are also available via figshare (See Data availability for more details).

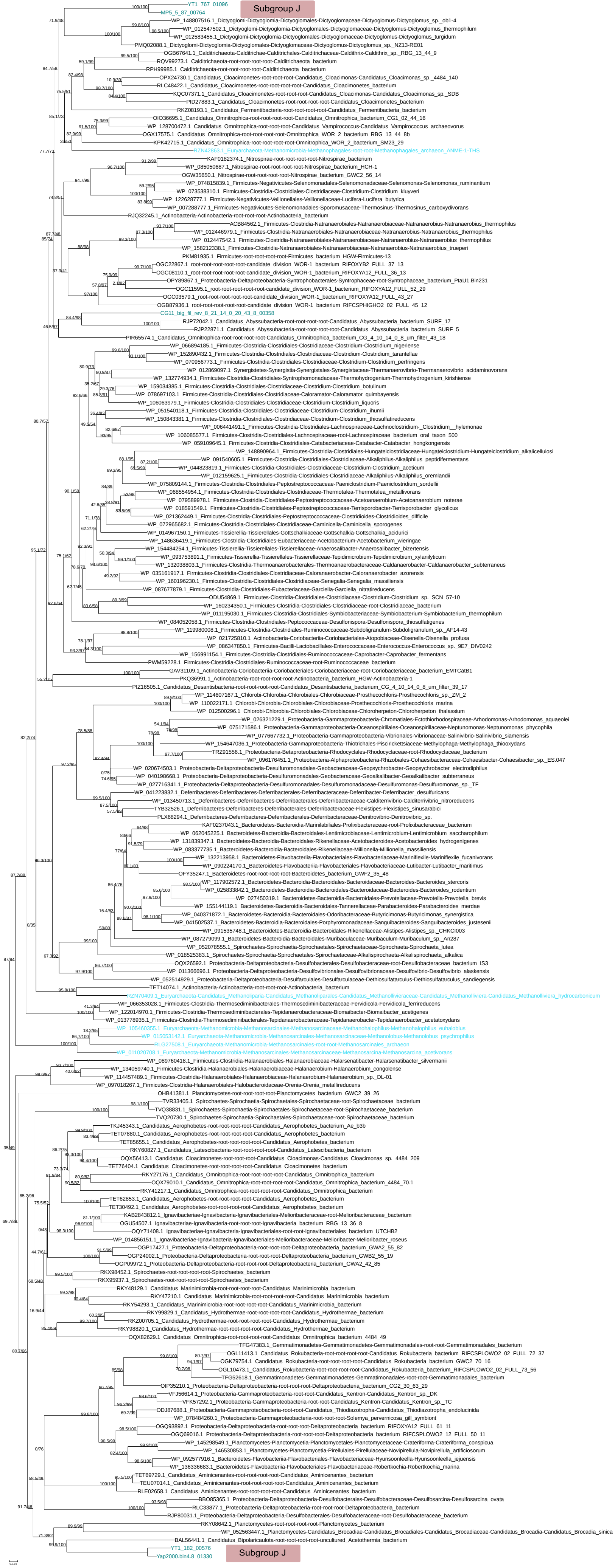

Supplementary Fig. 12: Phylogenetic analysis of *rnfG* gene (*Rhodobacter* nitrogen fixation complex subunit G). The tree was unrooted and inferred using IQ-Tree LG+C20+G model on an alignment of 192 taxa and 231 sites. UFBOOT (left) and SH-aLRT (right) support values are shown along the branches. Bacteria, Archaea and Woesearchaeota are colored in black, cyan, and green, respectively. Scale bar shows the number of average substitutions per site. Source data are provided as a Source Data file and raw data are also available via figshare (See Data availability for more details).

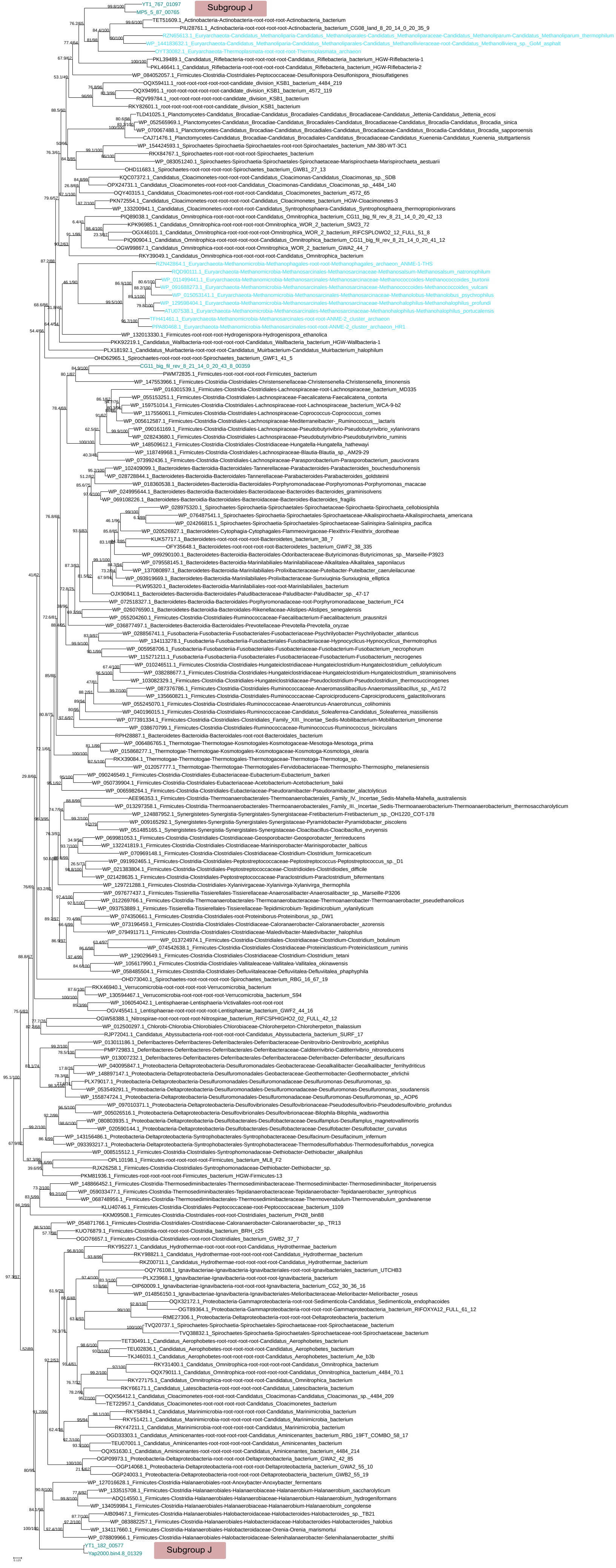

Supplementary Fig. 13: Phylogenetic analysis of *rnfE* gene (*Rhodobacter* nitrogen fixation complex subunit E). The tree was unrooted and inferred using IQ-Tree LG+C20+G model on an alignment of 198 taxa and 233 sites. UFBOOT (left) and SH-aLRT (right) support values are shown along the branches. Bacteria, Archaea and Woesearchaeota are colored in black, cyan, and green, respectively. Scale bar shows the number of average substitutions per site. Source data are provided as a Source Data file and raw data are also available via figshare (See Data availability for more details).

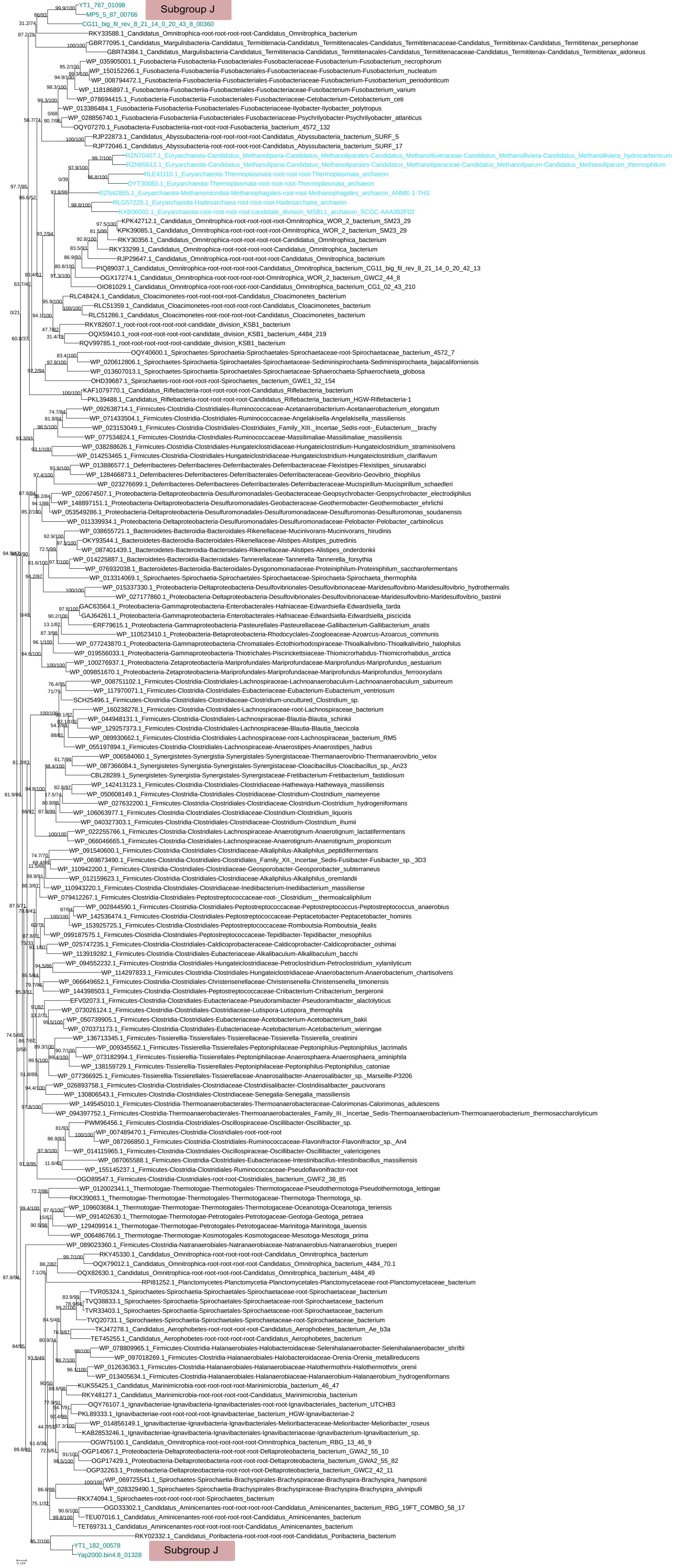

Supplementary Fig. 14: Phylogenetic analysis of *rnfA* gene (*Rhodobacter* nitrogen fixation complex subunit A). The tree was unrooted and inferred using IQ-Tree LG+C20+G model on an alignment of 166 taxa and 186 sites. UFBOOT (left) and SH-aLRT (right) support values are shown along the branches. Bacteria, Archaea and Woesearchaeota are colored in black, cyan, and green, respectively. Scale bar shows the number of average substitutions per site. Source data are provided as a Source Data file and raw data are also available via figshare (See Data availability for more details).

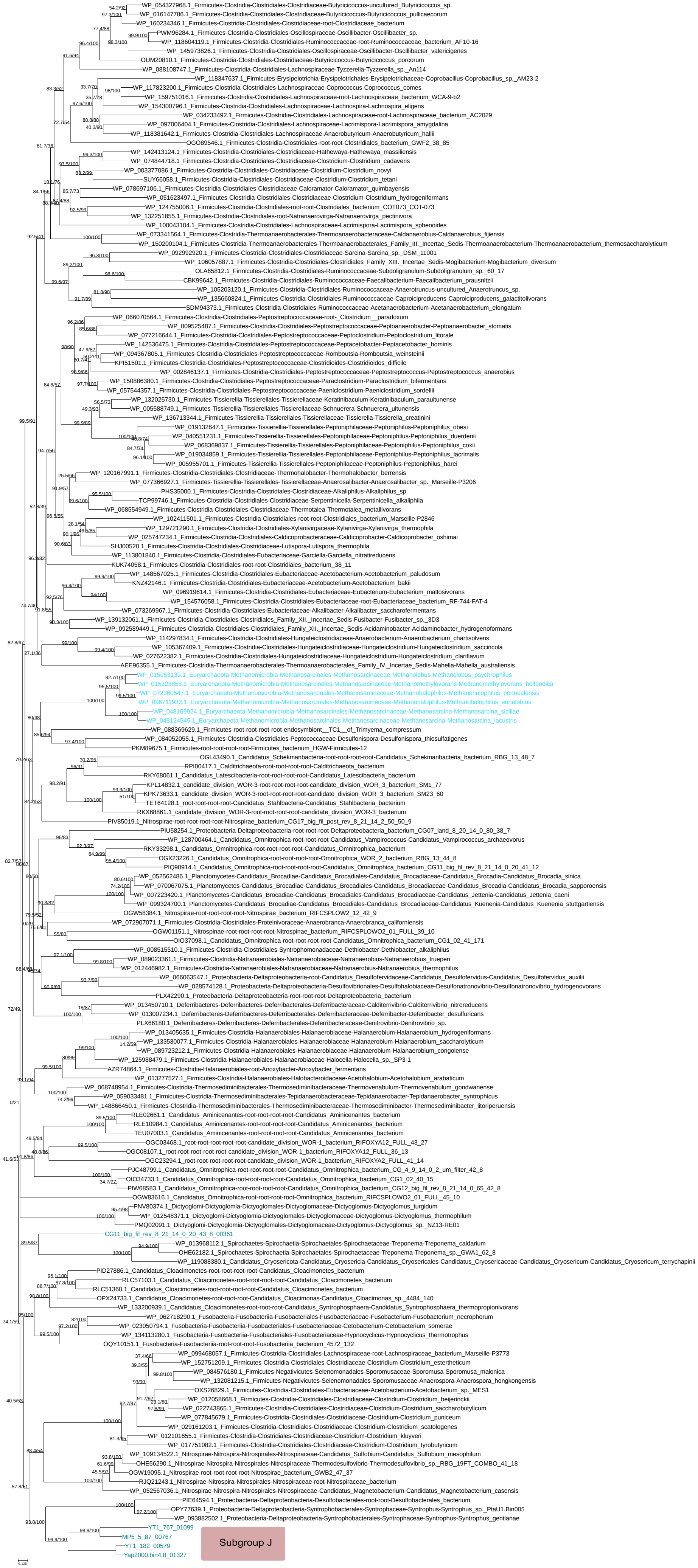

Supplementary Fig. 15: Phylogenetic analysis of *rnfB* gene (*Rhodobacter* nitrogen fixation complex subunit B). The tree was unrooted and inferred using IQ-Tree LG+C20+G model on an alignment of 170 taxa and 330 sites. UFBOOT (left) and SH-aLRT (right) support values are shown along the branches. Bacteria, Archaea and Woesearchaeota are colored in black, cyan, and green, respectively. Scale bar shows the number of average substitutions per site. Source data are provided as a Source Data file and raw data are also available via figshare (See Data availability for more details).

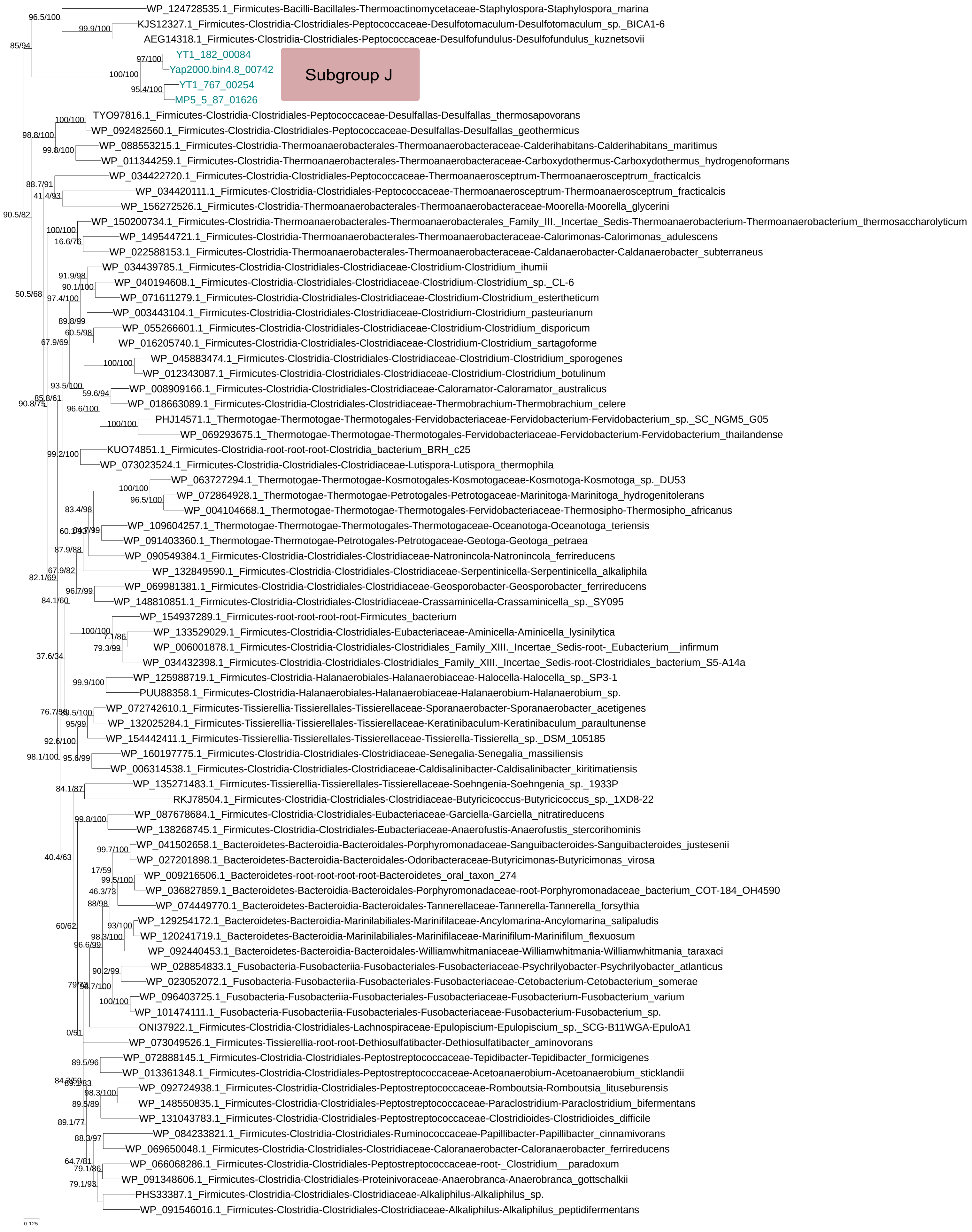

Supplementary Fig. 16: Phylogenetic analysis of *bcd* gene (butyryl-CoA dehydrogenase). The tree was unrooted and inferred using IQ-Tree LG +C20+G model on an alignment of 80 taxa and 356 sites. UFBOOT (left) and SH-aLRT (right) support values are shown along the branches. Bacteria, and Woesearchaeota are colored in black and green respectively. Scale bar shows the number of average substitutions per site. Source data are provided as a Source Data file and raw data are also available via figshare (See Data availability for more details).

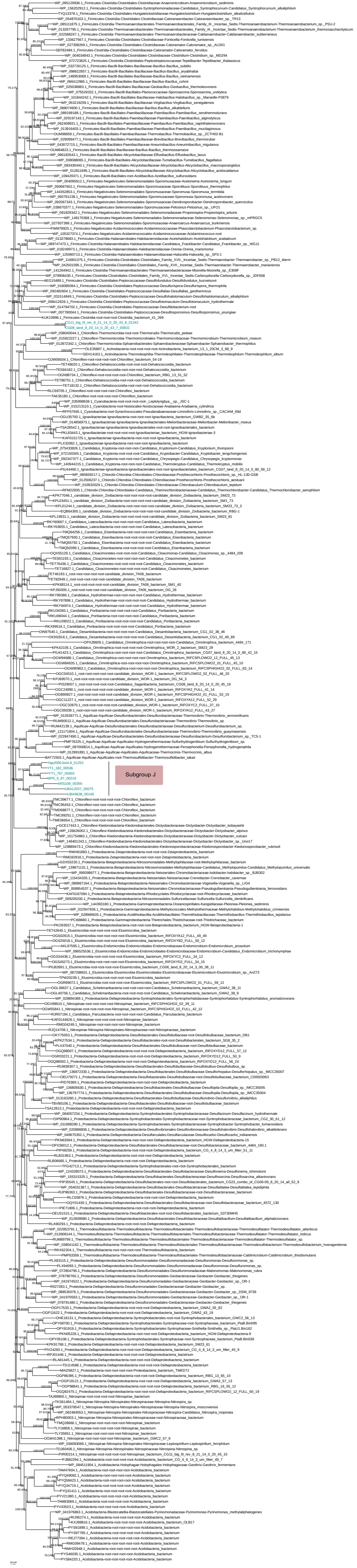

Supplementary Fig. 17: Phylogenetic analysis of *dxs* gene (1-deoxy-D-xylulose-5-phosphate synthase). The tree was unrooted and inferred using IQ-Tree LG+C50+F+I+G model on an alignment of 298 taxa and 695 sites. UFBOOT (left) and SH-aLRT (right) support values are shown along the branches. Bacteria and Woesearchaeota are colored in black and green respectively. Scale bar shows the number of average substitutions per site. Woesearchaeota subgroup J homologues are annotated in the figure. Source data are provided as a Source Data file and raw data are also available via figshare (See Data availability for more details).

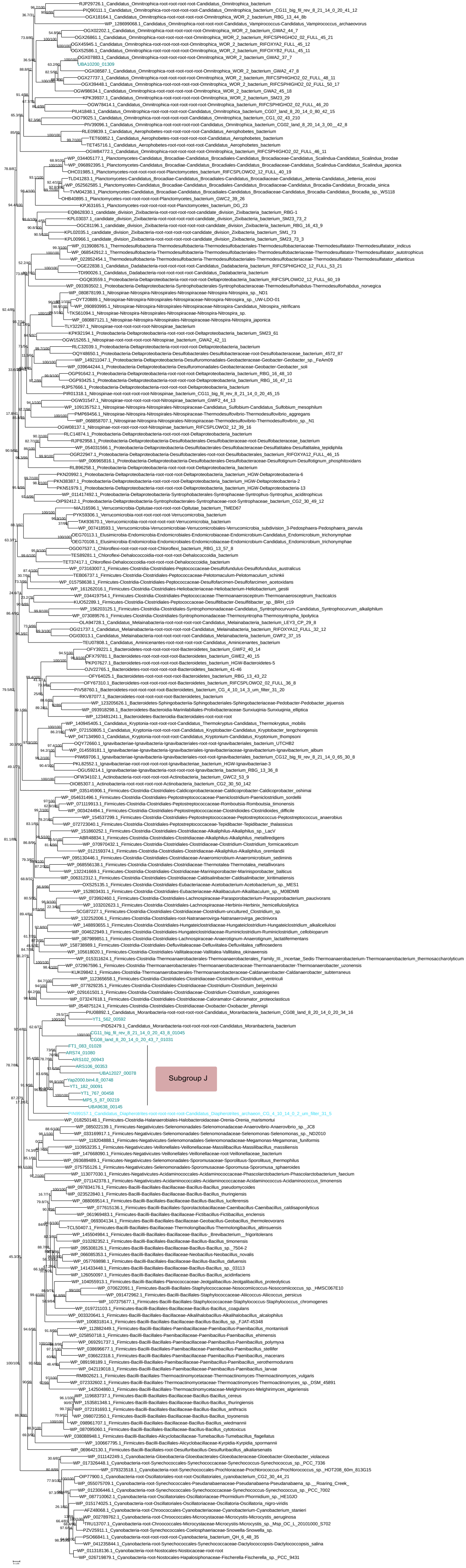

Supplementary Fig. 18: Phylogenetic analysis of *dxr* gene (1-deoxy-D-xylulose 5-phosphate reductoisomerase). The tree was unrooted and inferred using IQ-Tree LG+C60+F+I+G model on an alignment of 232 taxa and 403 sites. UFBOOT (left) and SH-aLRT (right) support values are shown along the branches. Scale bar shows the number of average substitutions per site. Bacteria, Archaea and Woesearchaeota are colored in black, cyan andgreen respectively. Woesearchaeota subgroup J is annotated in the figure. Source data are provided as a Source Data file and raw data are also available via figshare (See Data availability for more details).

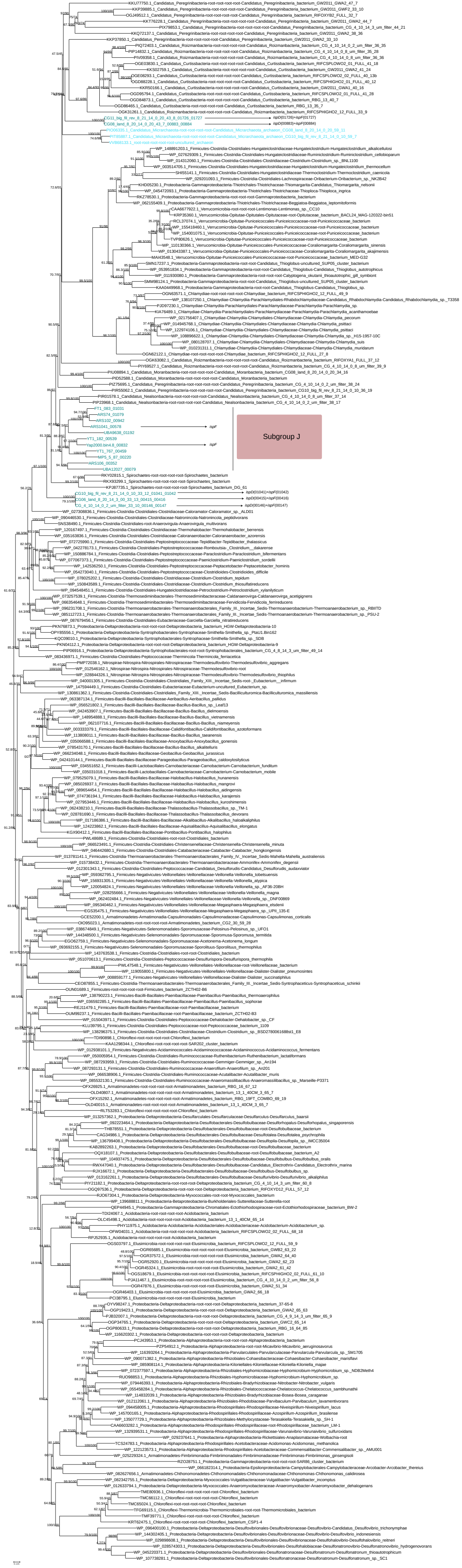

Supplementary Fig. 19: Phylogenetic analysis of *ispD/F* gene (2-C-methyl-D-erythritol 4-phosphate cytidylyltransferase/2-C-methyl-D-erythritol 2,4 cyclodiphosphate synthase). The tree was unrooted and inferred using IQ-Tree LG+C20+F+G model on an alignment of 258 taxa and 465 sites. UFBOOT (left) and SH-aLRT (right) support values are shown along the branches. Bacteria, Archaea and Woesearchaeota are colored in black, cyan and green respectively. Scale bar shows the number of average substitutions per site. Woesearchaeota subgroup J homologues are annotated in the figure. Source data are provided as a Source Data file and raw data are also available via figshare (See Data availability for more details).

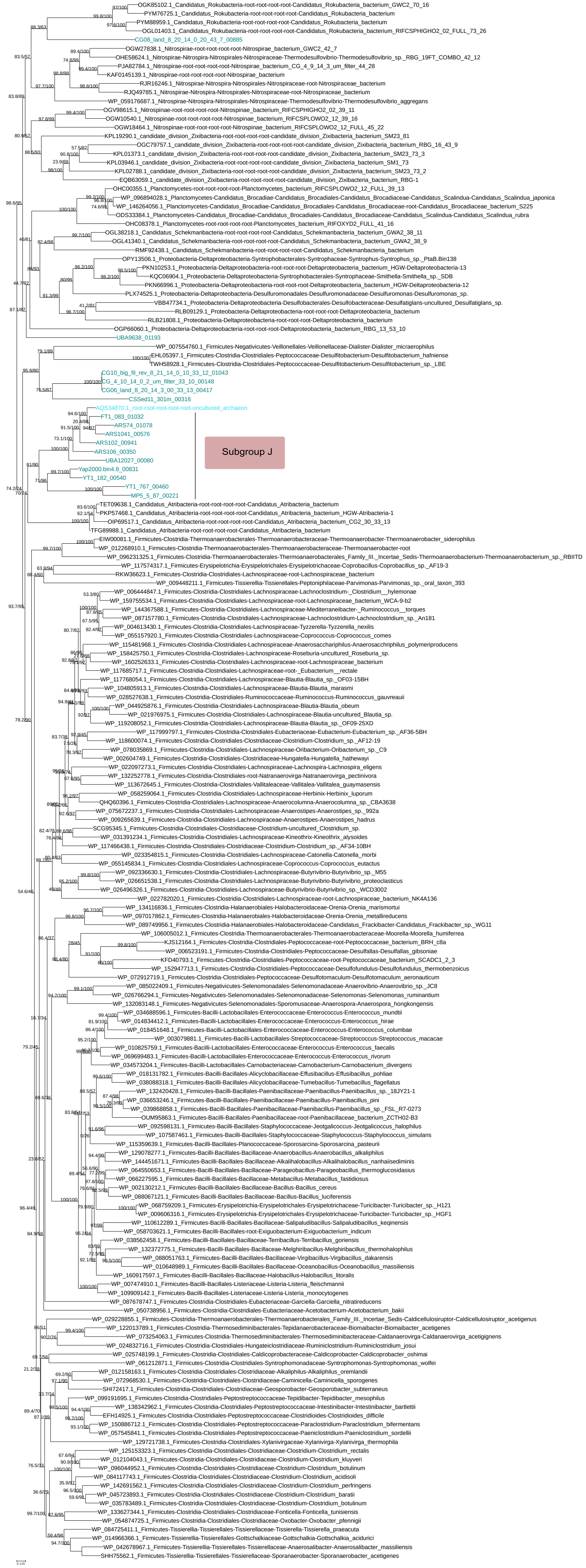

Supplementary Fig. 20: Phylogenetic analysis of *ispE* gene (4-diphosphocytidyl-2-C-methyl-D-erythritol kinase). The tree was unrooted and inferred using IQ-Tree LG+C60+F+I+G model on an alignment of 178 taxa and 322 sites. UFBOOT (left) and SH-aLRT (right) support values are shown along the branches. Bacteria, Archaea and Woesearchaeota are colored in black, cyan and green respectively. Scale bar shows the number of average substitutions per site. Woesearchaeota subgroup J homologues are annotated in the figure. Source data are provided as a Source Data file and raw data are also available via figshare (See Data availability for more details).

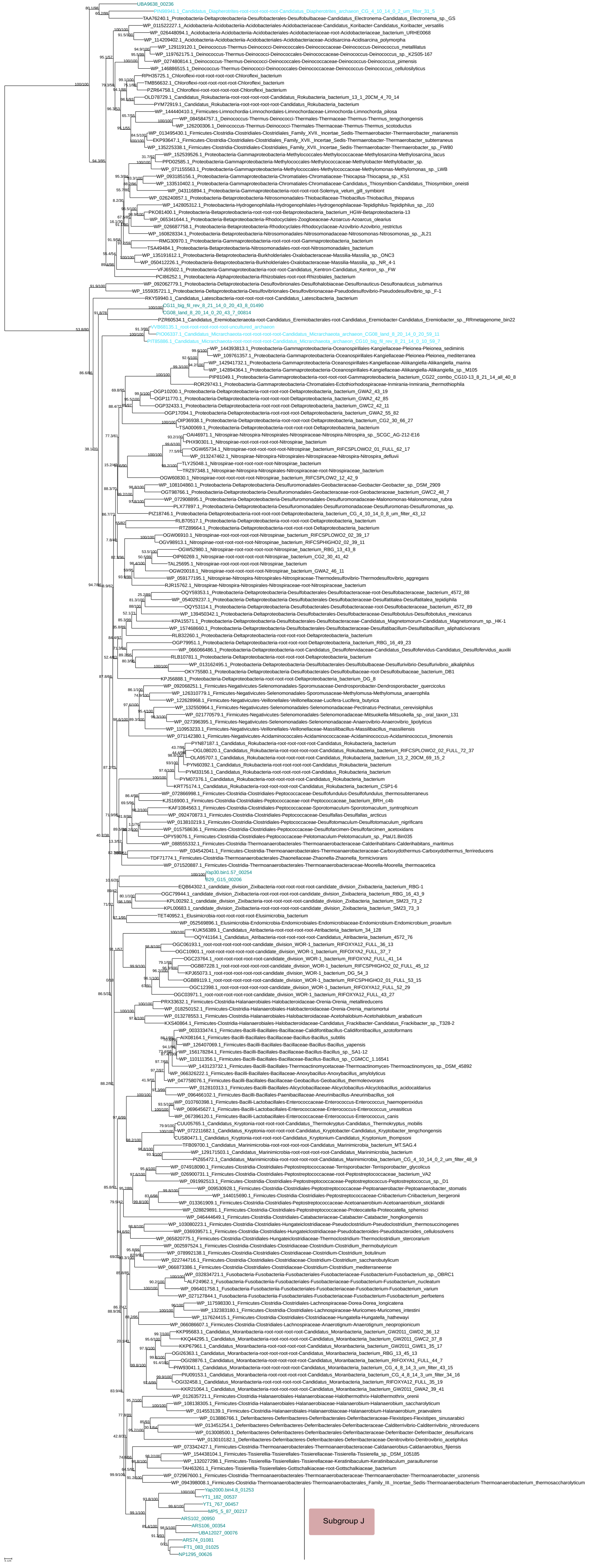

Supplementary Fig. 21: Phylogenetic analysis of *ispG* gene (4-hydroxy-3-methylbut-2-en-1-yl diphosphate synthase). The tree was unrooted and inferred using IQ-Tree LG+C20+F+G model on an alignment of 217 taxa and 445 sites. UFBOOT (left) and SH-aLRT (right) support values are shown along the branches. Bacteria, Archaea and Woesearchaeota are colored in black, cyan and green respectively. Woesearchaeota subgroup J homologues are annotated in the figure. Scale bar shows the number of average substitutions per site. Source data are provided as a Source Data file and raw data are also available via figshare (See Data availability for more details).

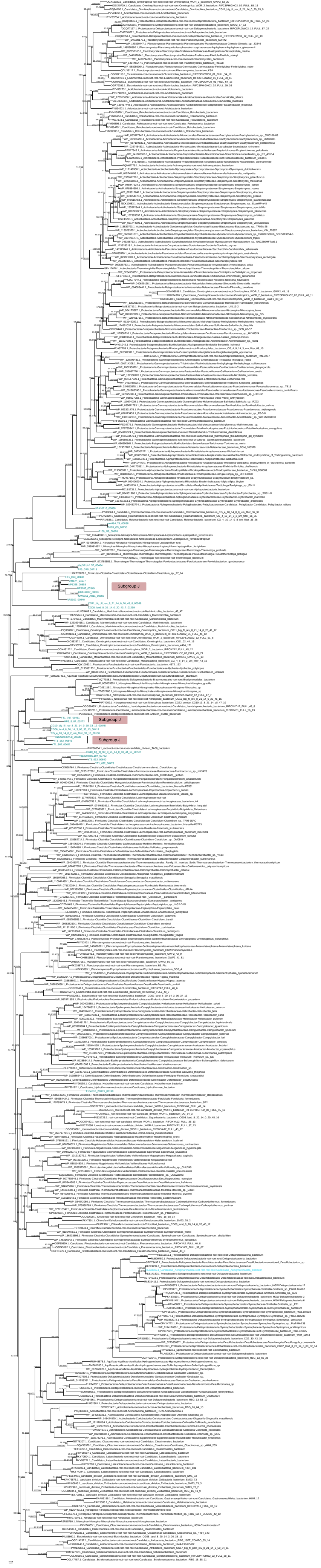

Supplementary Fig. 22: Phylogenetic analysis of *ispH* gene (4-hydroxy-3-methylbut-2-enyl diphosphate reductase). The tree was unrooted and inferred using IQ-Tree LG+C60+F+I+G model on an alignment of 410 taxa and 856 sites. UFBOOT (left) and SH-aLRT (right) support values are shown along the branches. Bacteria, Archaea and Woesearchaeota are colored in black, cyan and green, respectively. Scale bar shows the number of average substitutions per site. Woesearchaeota subgroup J homologues are annotated in the figure. Source data are provided as a Source Data file and raw data are also available via figshare (See Data availability for more details).

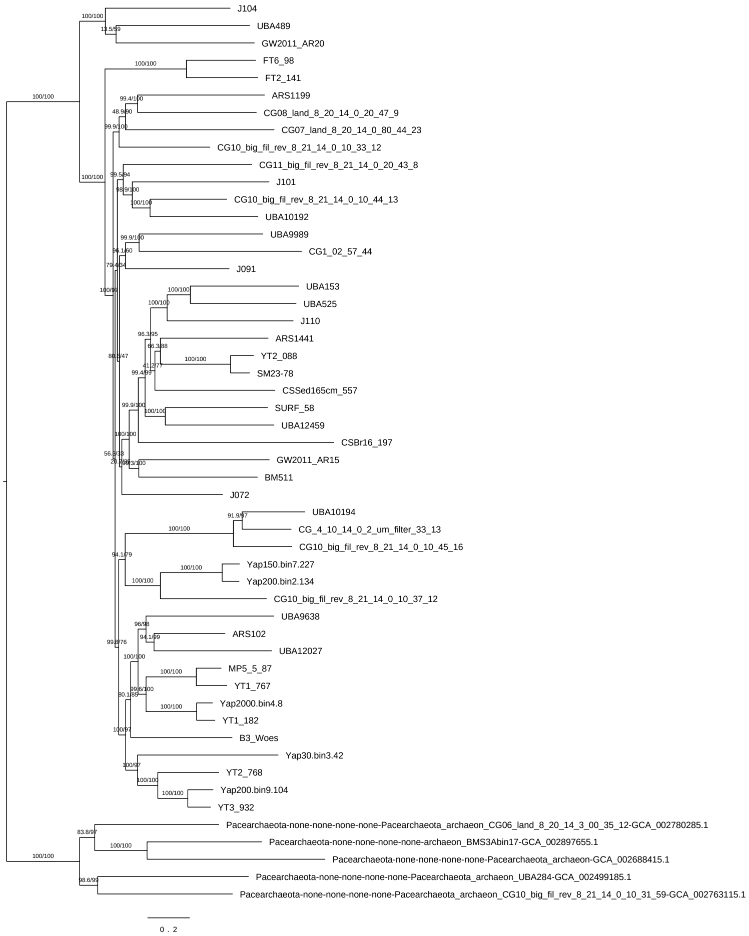

Supplementary Fig. 23: Full tree used in Fig. 5a. The tree is rooted using Pacearchaeota and based on an alignment of 52 taxa and 10896 sites. Raw data are available via figshare (see Data availability for more details). Scale bar shows the number of average substitutions per site.

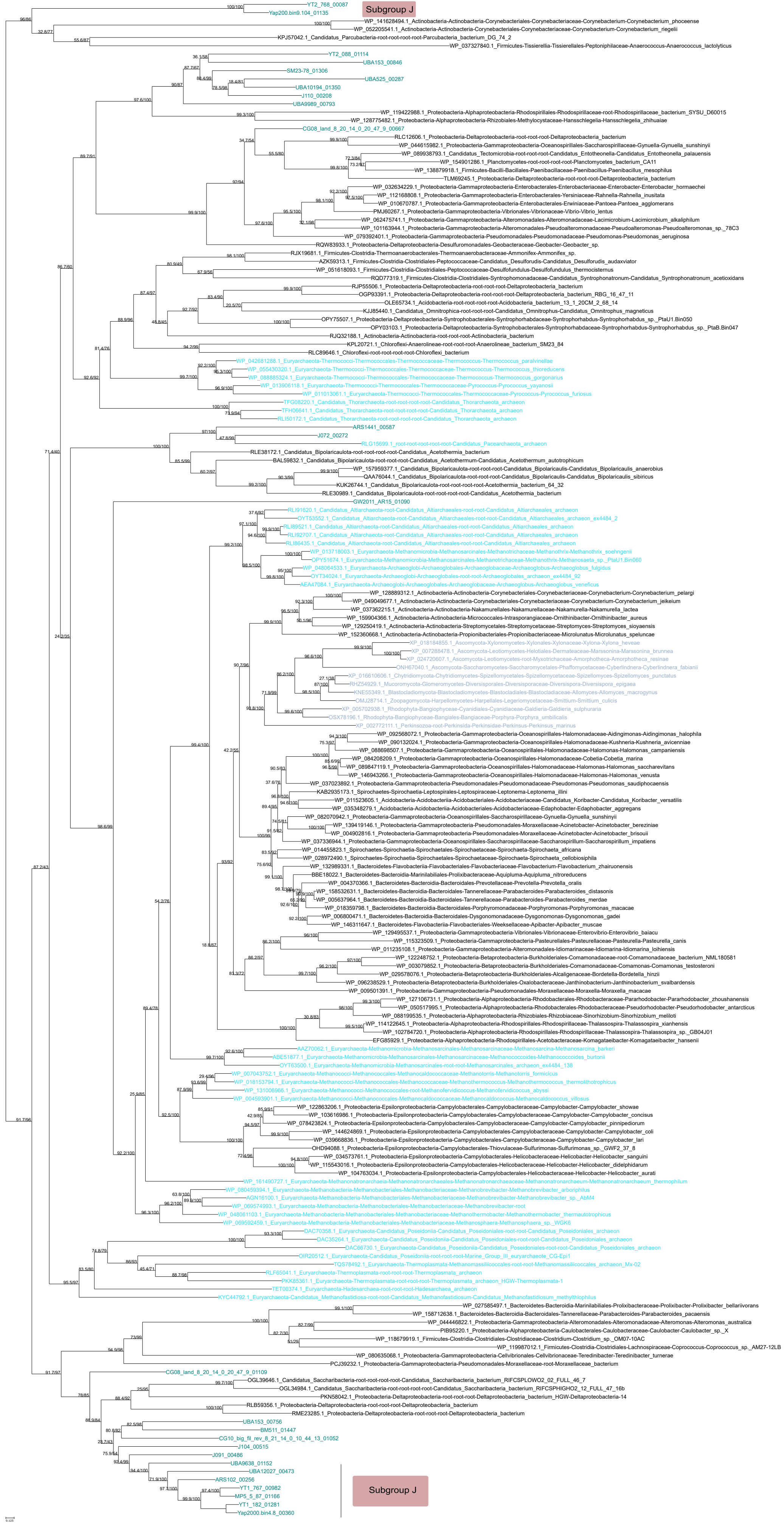

Supplementary Fig. 24: Phylogenetic analysis of *serB* gene (Phosphoserine phosphatase). The tree was unrooted and inferred using IQ-Tree LG+C20+G model on an alignment of 183 taxa and 430 sites. UFBOOT (left) and SH-aLRT (right) support values are shown along the branches. Bacteria, Archaea, Woesearchaeota and Eukaryotes are colored in black, cyan, green and gray respectively. Woesearchaeota sequences are selected from 47 MAGs used in the inference of proteome content change history. Woesearchaeota subgroup J homologues are annotated in the figure. Scale bar shows the number of average substitutions per site. Source data are provided as a Source Data file and raw data are also available via figshare (See Data availability for more details).

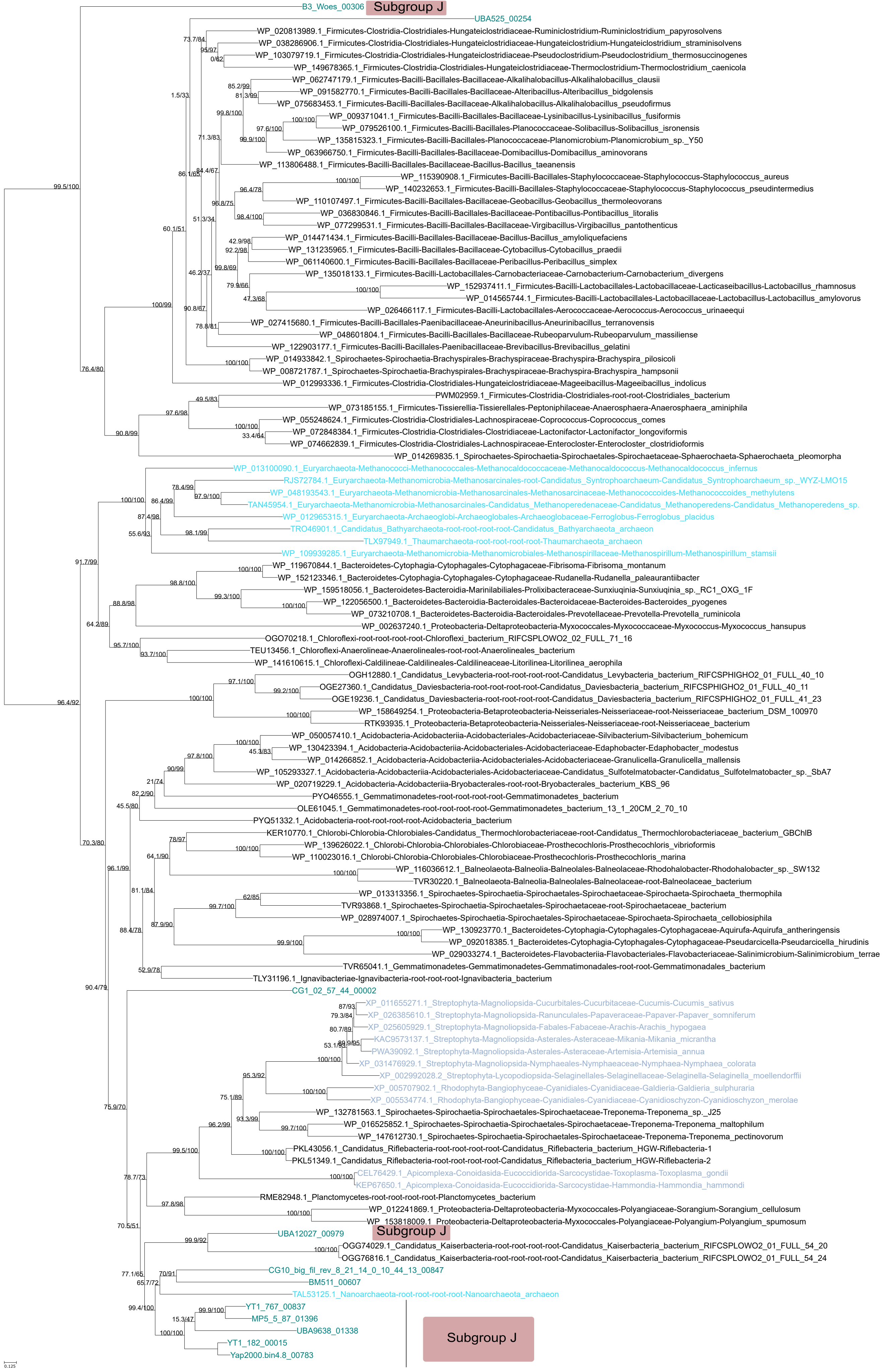

Supplementary Fig. 25: Phylogenetic analysis of *lysC* gene (aspartokinase). The tree was unrooted and inferred using IQ-Tree LG+C20+G model on an alignment of 112 taxa and 507 sites. UFBOOT (left) and SH-aLRT (right) support values are shown along the branches. Bacteria, Archaea, Woesearchaeota and Eukaryotes are colored in black, cyan, green and grey respectively. Woesearchaeota sequences are selected from 47 MAGs used in the inference of proteome content change history. Scale bar shows the number of average substitutions per site. Source data are provided as a Source Data file and raw data are also available via figshare (See Data availability for more details).

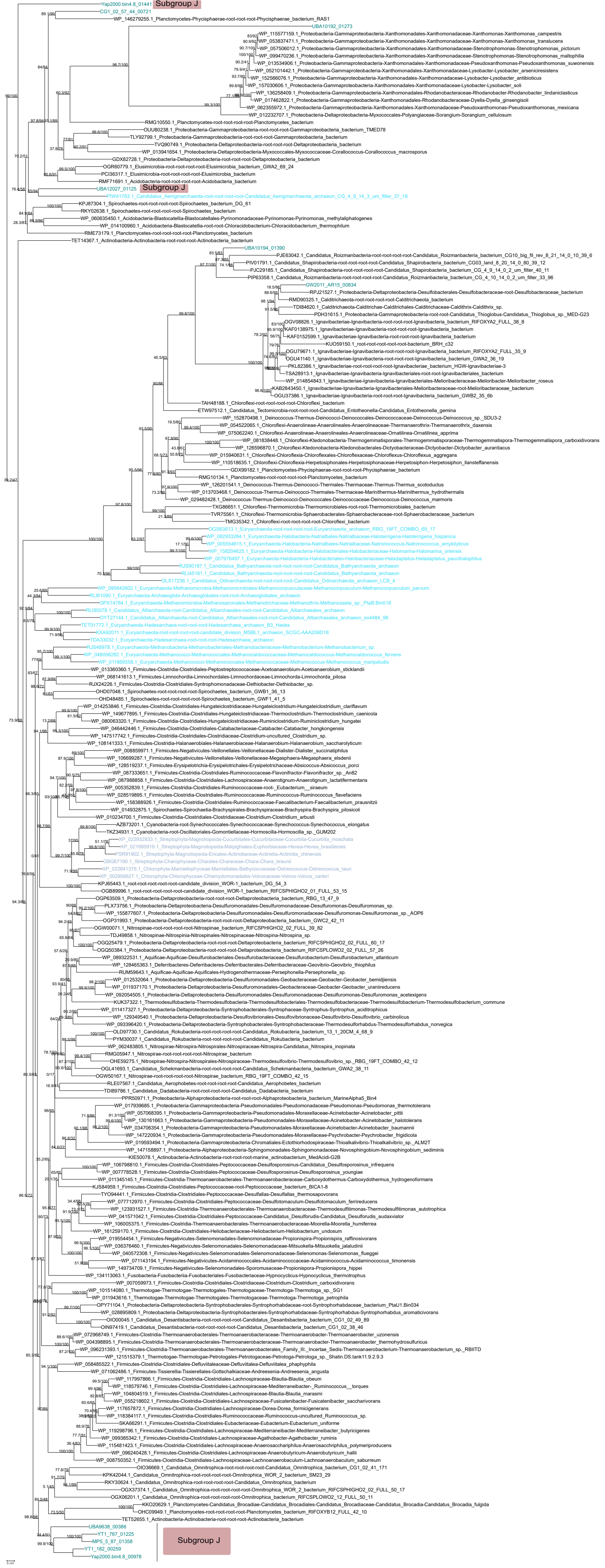

Supplementary Fig. 26: Phylogenetic analysis of *argB* gene (acetylglutamate kinase). The tree was unrooted and inferred using IQ-Tree LG+C20 +G model on an alignment of 211 taxa and 319 sites. UFBOOT (left) and SH-aLRT (right) support values are shown along the branches. Bacteria, Archaea, Woesearchaeota and Eukaryotes are colored in black, cyan, green and grey, respectively. Woesearchaeota sequences are selected from 47 MAGs used in the inference of proteome content change history. Scale bar shows the number of average substitutions per site. Woesearchaeota subgroup J homologues are annotated in the figure. Source data are provided as a Source Data file and raw data are also available via figshare (See Data availability for more details).

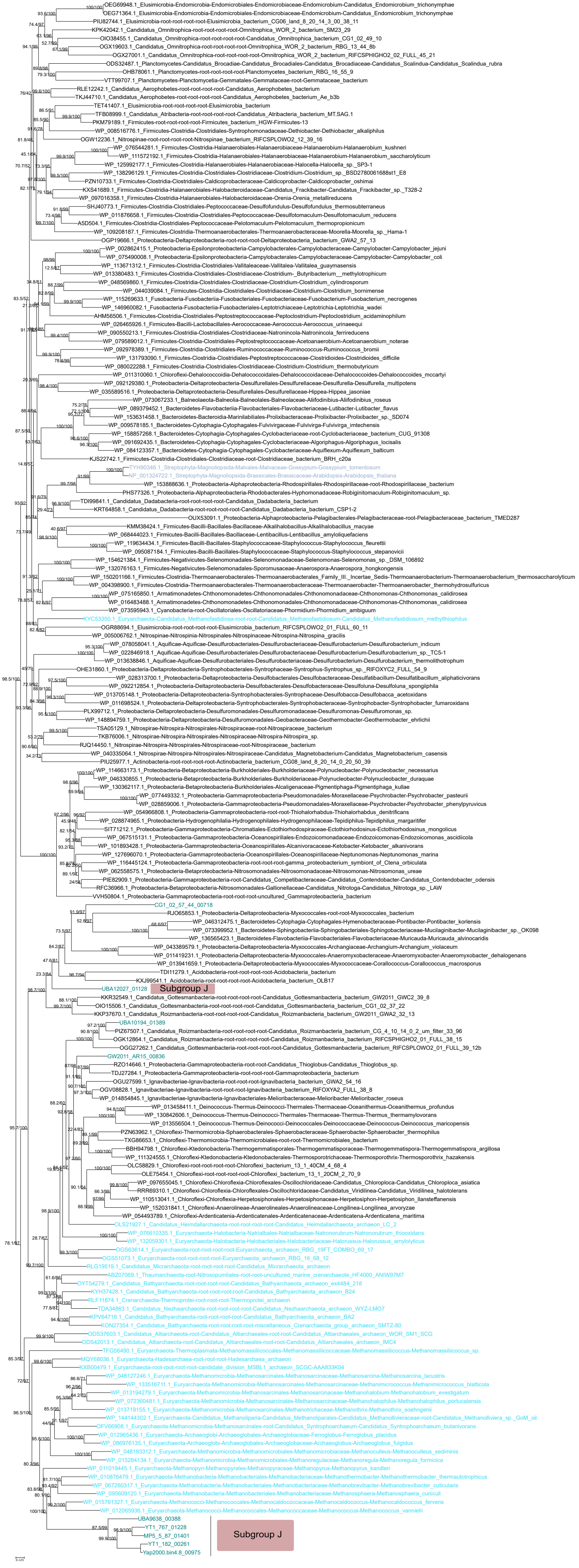

Supplementary Fig. 27: Phylogenetic analysis of *argC* gene (N-acetyl-gamma-glutamyl-phosphate reductase). The tree was unrooted and inferred using IQ-Tree LG+C20+G model on an alignment of 185 taxa and 351 sites. UFBOOT (left) and SH-aLRT (right) support values are shown along the branches. Bacteria, Archaea, Woesearchaeota and Eukaryotes are colored in black, cyan, green and grey respectively. Woesearchaeota sequences are selected from 47 MAGs used in the inference of proteome content change history. Scale bar shows the number of average substitutions per site. Woesearchaeota subgroup J homologues are annotated in the figure. Source data are provided as a Source Data file and raw data are also available via figshare (See Data availability for more details).

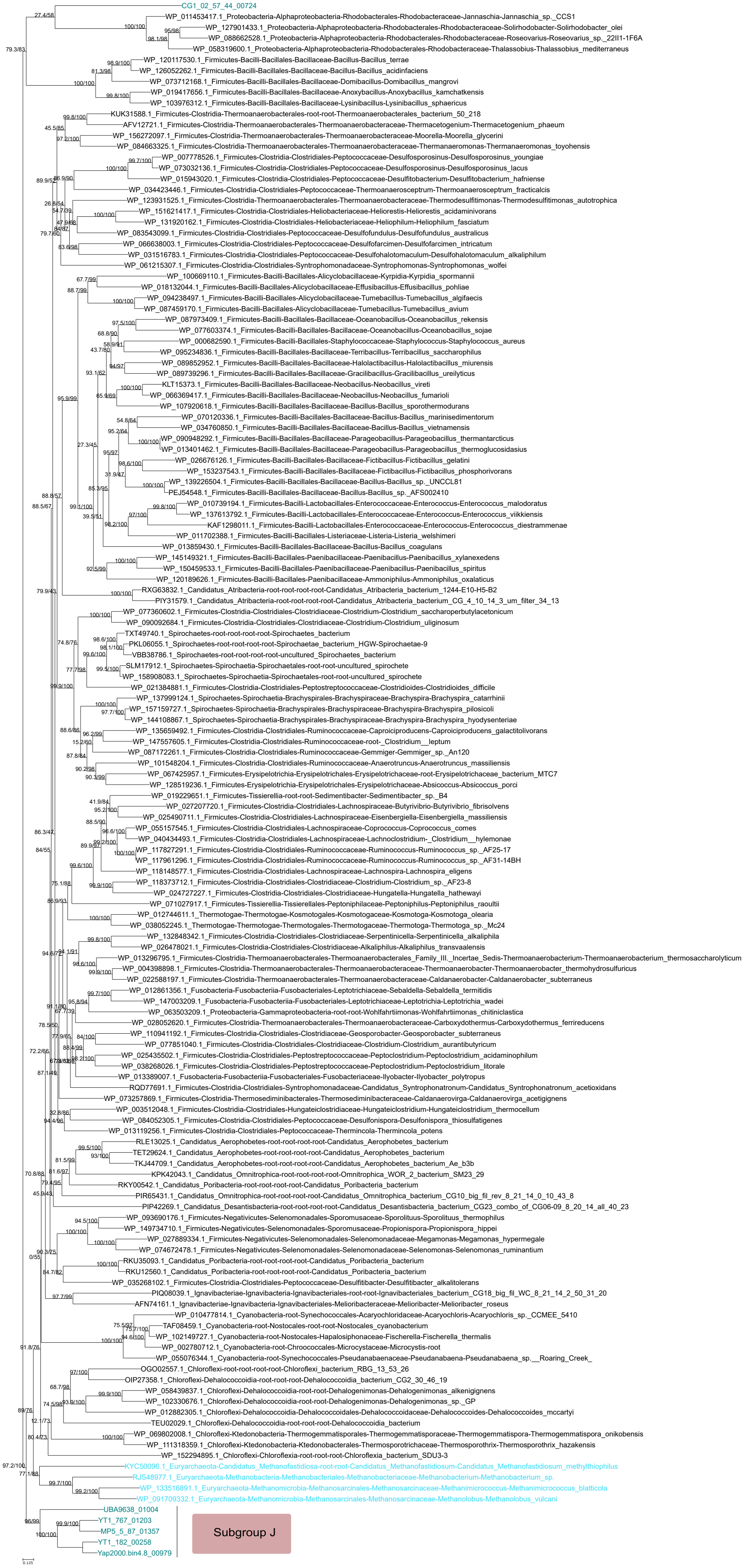

Supplementary Fig. 28: Phylogenetic analysis of *argJ* gene (glutamate N-acetyltransferase/amino-acid acetyltransferase). The tree was unrooted and inferred using IQ-Tree LG+C20+G model on an alignment of 144 taxa and 402 sites. UFBOOT (left) and SH-aLRT (right) support values are shown along the branches. Bacteria, Archaea and Woesearchaeota are colored in black, cyan and green, respectively. Woesearchaeota sequences are selected from 47 MAGs used in the inference of proteome content change history. Woesearchaeota subgroup J homologues are annotated in the figure. Scale bar shows the number of average substitutions per site. Source data are provided as a Source Data file and raw data are also available via figshare (See Data availability for more details).

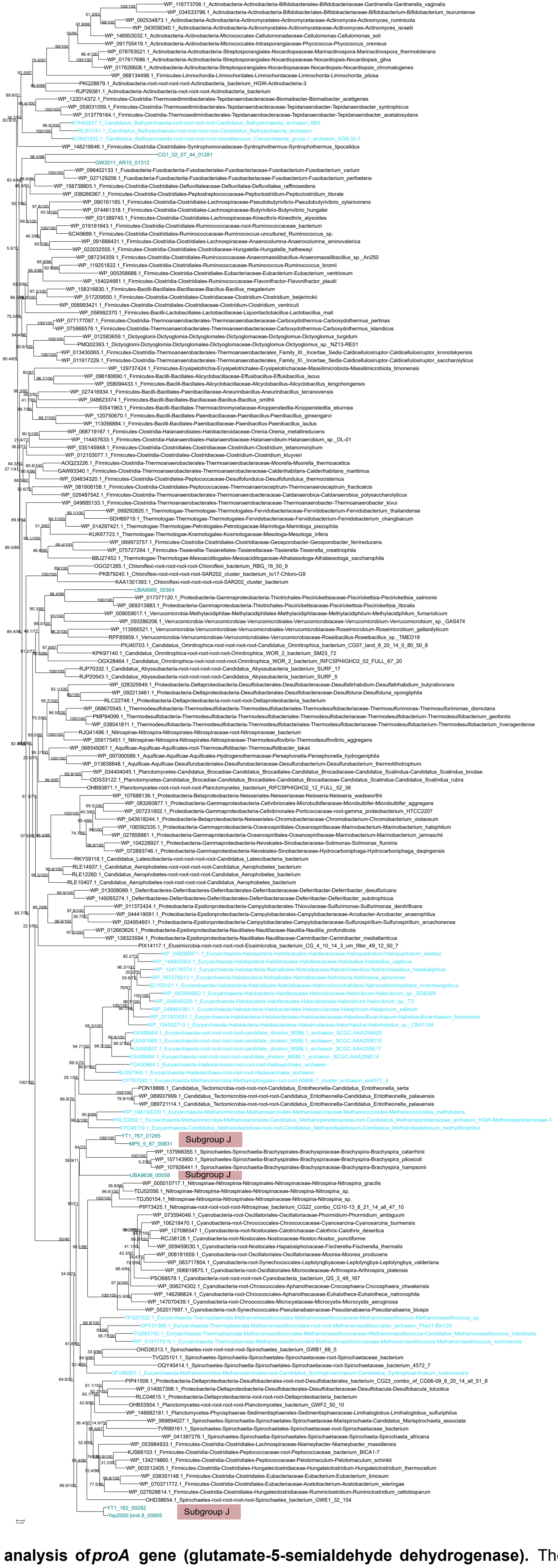

Supplementary Fig. 29: Phylogenetic analysis of *proA* gene (glutamate-5-semialdehyde dehydrogenase). The tree was unrooted and inferred using IQ-Tree LG+C20+G model on an alignment of 192 taxa and 444 sites. UFBOOT (left) and SH-aLRT (right) support values are shown along the branches. Bacteria, Archaea and Woesearchaeota are colored in black, cyan, and green, respectively. Woesearchaeota sequences are selected from 47 MAGs used in the inference of proteome content change history. Woesearchaeota subgroup J homologues are annotated in the figure. Scale bar shows the number of average substitutions per site. Source data are provided as a Source Data file and raw data are also available via figshare (See Data availability for more details).

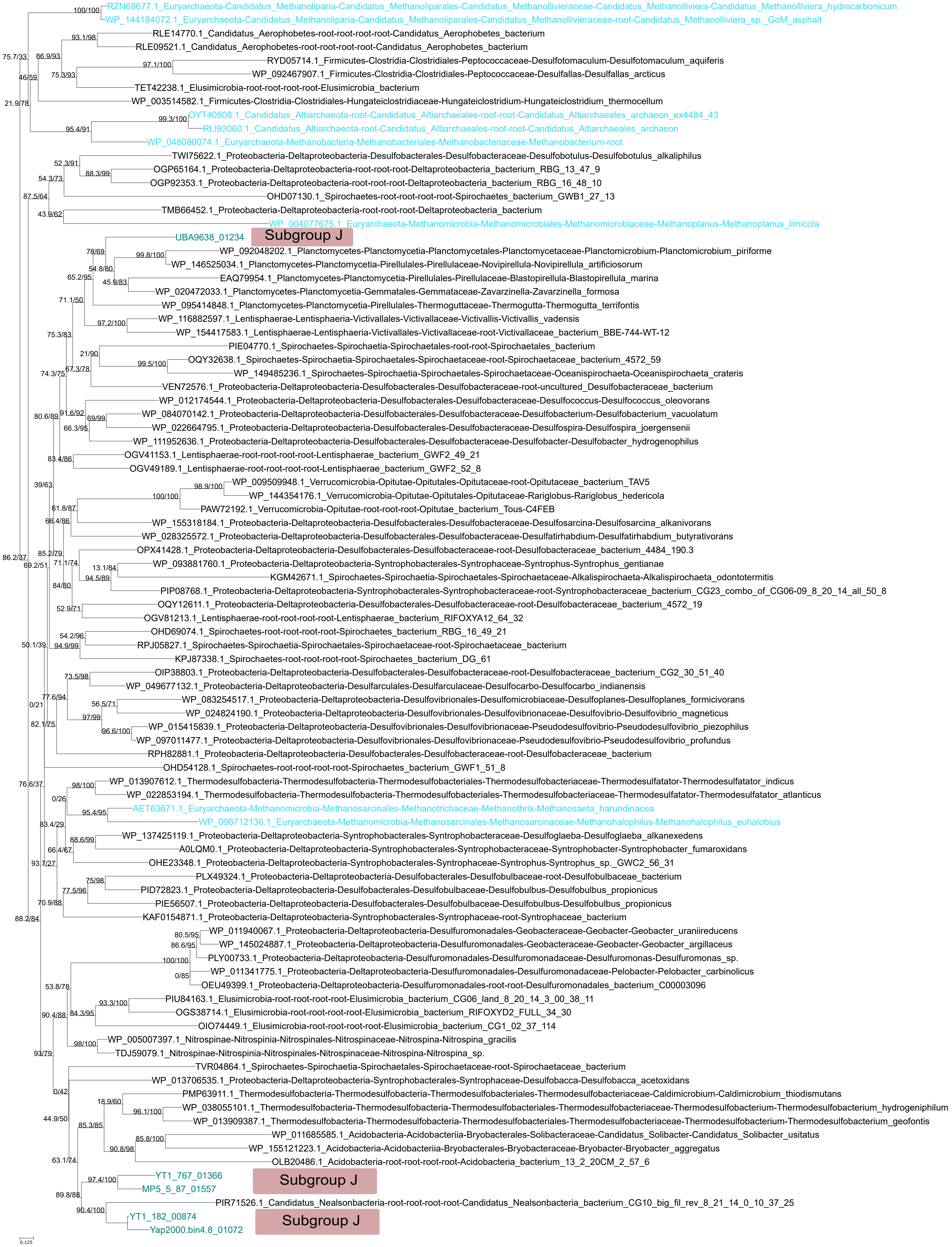

Supplementary Fig. 30: Phylogenetic analysis *of hisI* gene (Phosphoribosyl-AMP cyclohydrolase). The tree was unrooted and inferred using IQ-Tree LG+C20+G model on an alignment of 91 taxa and 128 sites. UFBOOT (left) and SH-aLRT (right) support values are shown along the branches. Bacteria, Archaea and Woesearchaeota are colored in black, cyan and green, respectively. Woesearchaeota sequences are selected from 47 MAGs used in the inference of proteome content change history. Scale bar shows the number of average substitutions per site. Woesearchaeota subgroup J homologues are annotated in the figure. Source data are provided as a Source Data file and raw data are also available via figshare (See Data availability for more details).

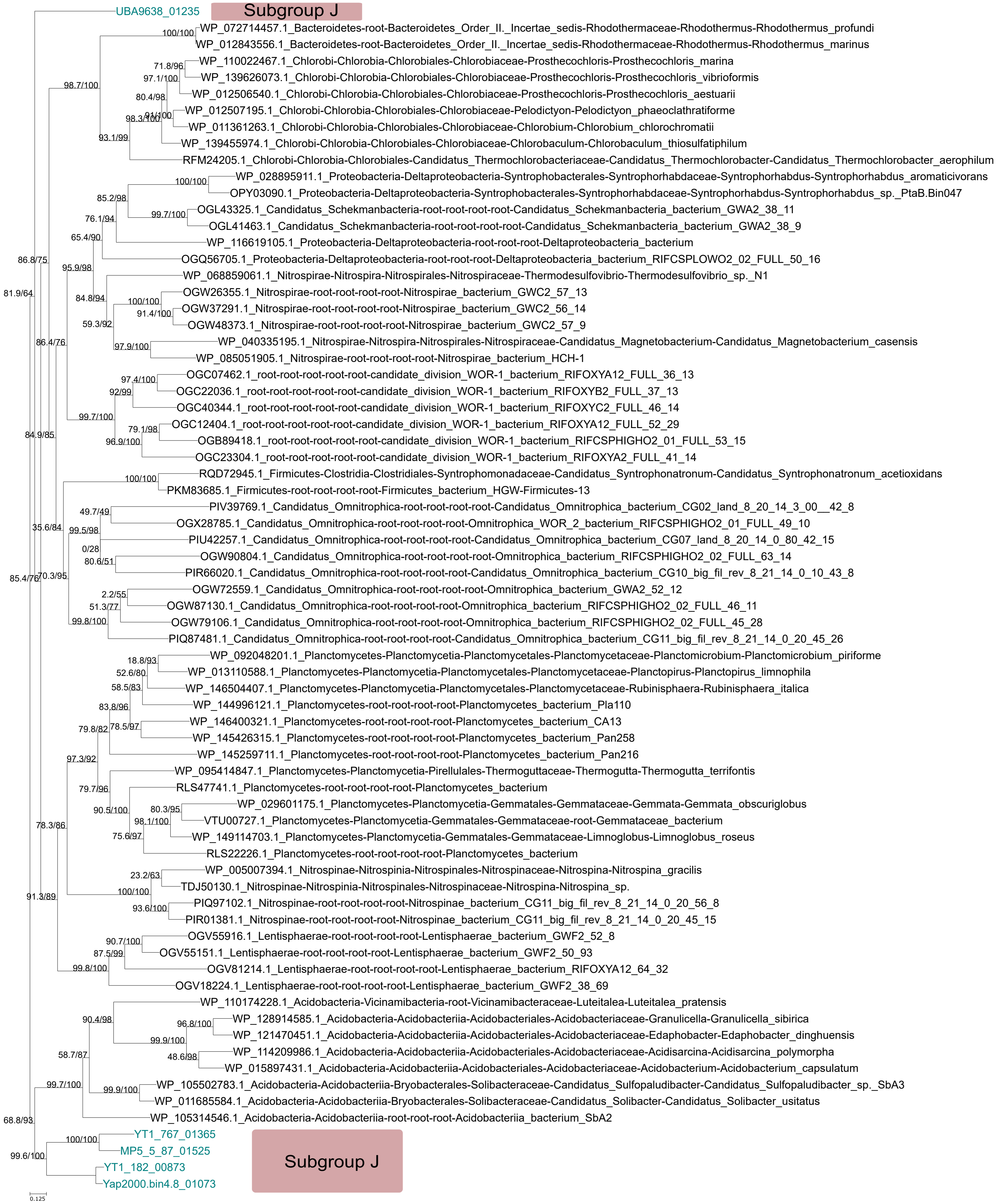

Supplementary Fig. 31: Phylogenetic analysis of *hisG* gene (ATP phosphoribosyltransferase). The tree was unrooted and inferred using IQ-Tree LG+C20+G model on an alignment of 72 taxa and 279 sites. UFBOOT (left) and SH-aLRT (right) support values are shown along the branches. Bacteria and Woesearchaeota are colored in black and green, respectively. Woesearchaeota sequences are selected from 47 MAGs used in the inference of proteome content change history. Woesearchaeota subgroup J homologues are annotated in the figure. Scale bar shows the number of average substitutions per site. Source data are provided as a Source Data file and raw data are also available via figshare (See Data availability for more details).

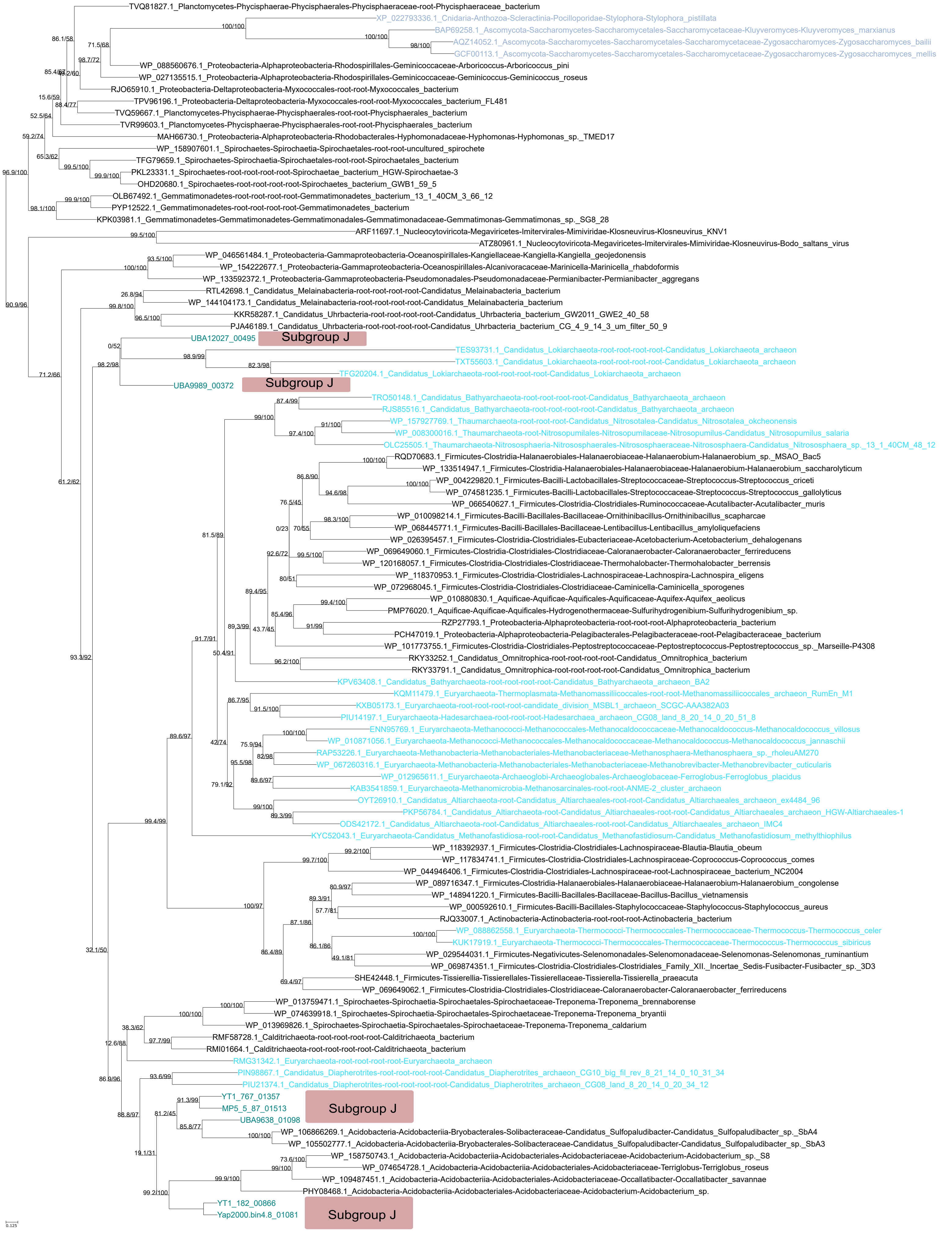

Supplementary Fig. 32: Phylogenetic analysis of *hisA* gene (phosphoribosylformimino-5-aminoimidazole carboxamide ribotide isomerase). The tree was unrooted and inferred using IQ-Tree LG+C20+G model on an alignment of 103 taxa and 449 sites. UFBOOT (left) and SH-aLRT (right) support values are shown along the branch. Bacteria, Archaea, Woesearchaeota and Eukaryotes are colored in black, cyan, green and grey, respectively. Woesearchaeota sequences are selected from 47 MAGs used in the inference of proteome content change history. Scale bar shows the number of average substitutions per site. Woesearchaeota subgroup J homologues are annotated in the figure. Source data are provided as a Source Data file and raw data are also available via figshare (See Data availability for more details).

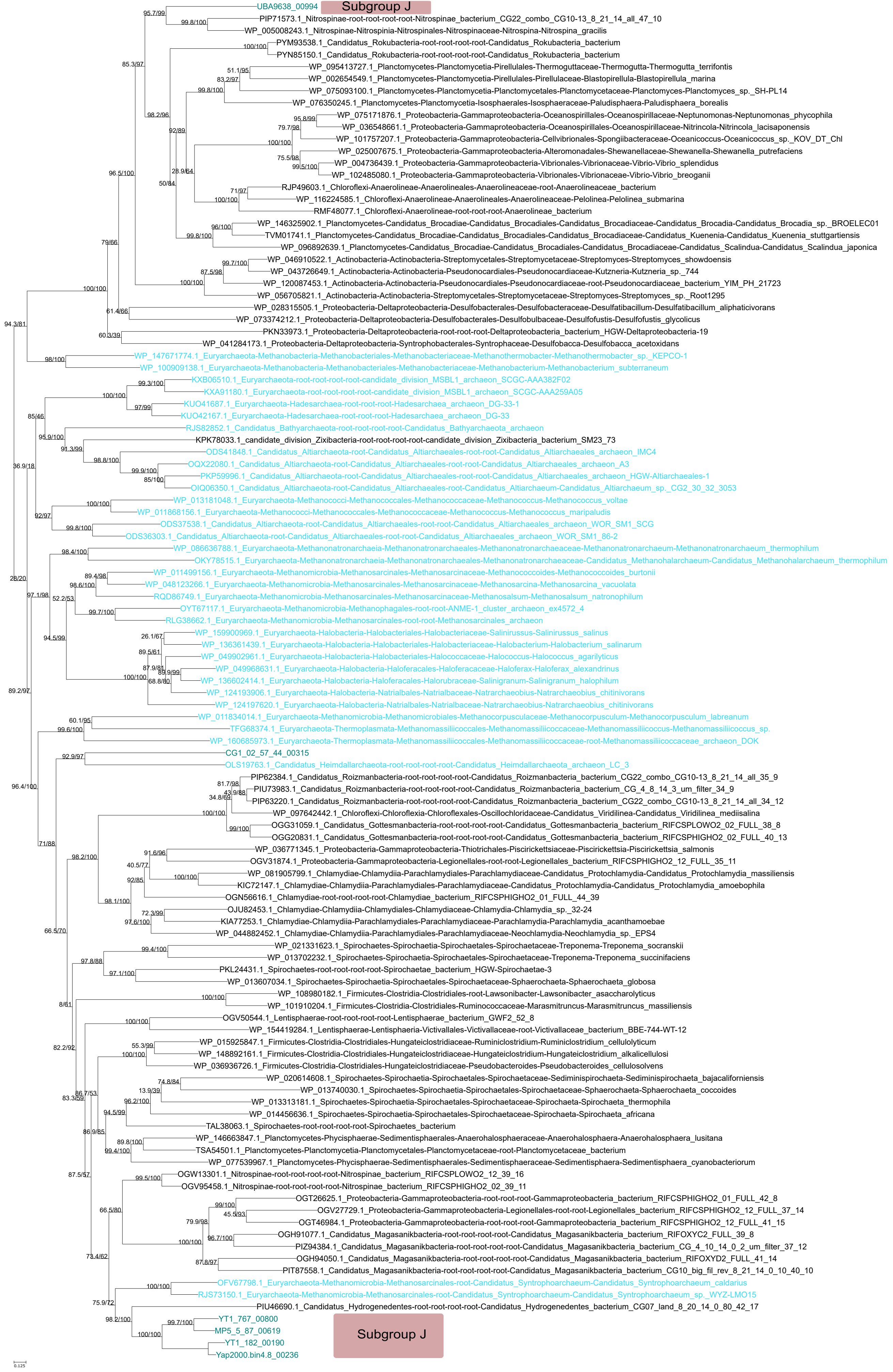

Supplementary Fig. 33: Phylogenetic analysis of *aroA* gene (3-phosphoshikimate 1-carboxyvinyltransferase). The tree was unrooted and inferred using IQ-Tree LG+C20+G model on an alignment of 113 taxa and 437 sites. UFBOOT (left) and SH-aLRT (right) support values are shown along the branches. Bacteria, Archaea and Woesearchaeota are colored in black, cyan and green, respectively. Woesearchaeota sequences are selected from 47 MAGs used in the inference of proteome content change history. Woesearchaeota subgroup J homologues are annotated in the figure. Scale bar shows the number of average substitutions per site. Source data are provided as a Source Data file and raw data are also available via figshare (See Data availability for more details).

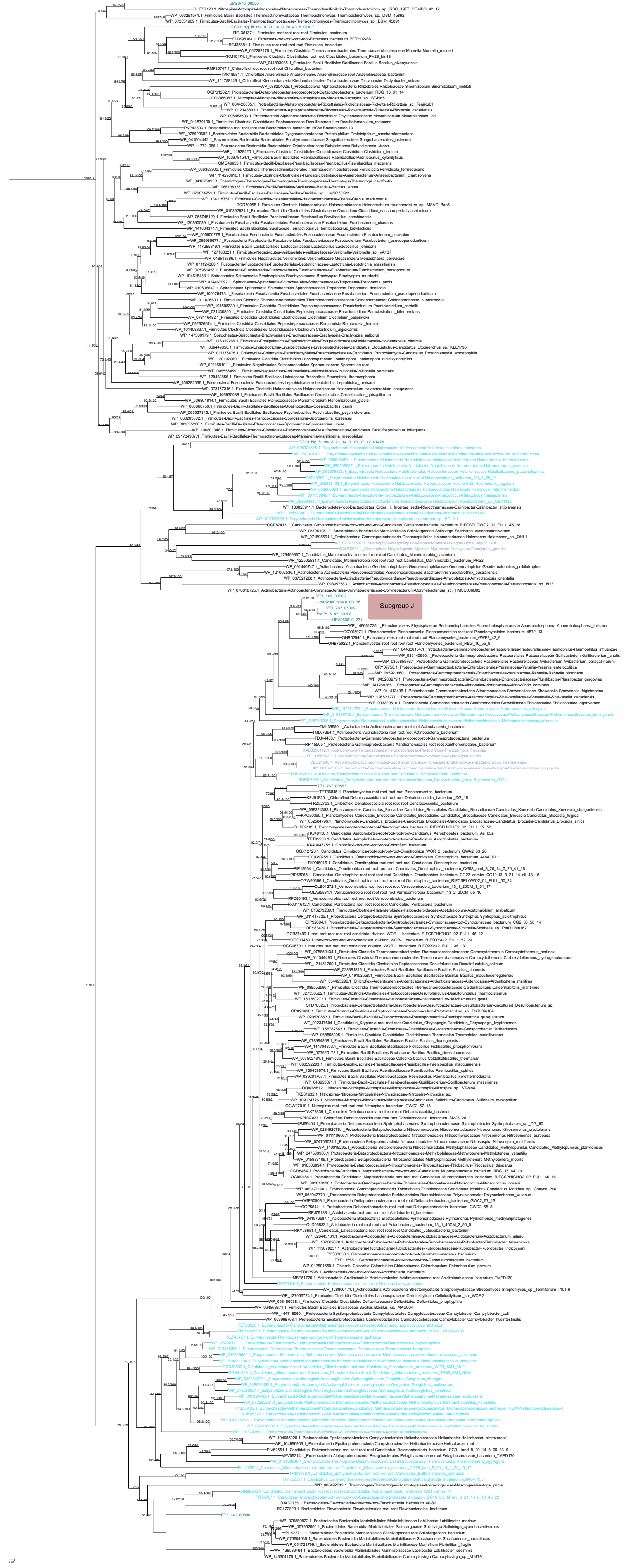

Supplementary Fig. 34: Phylogenetic analysis of *trpG* gene (glutamine amidotransferase of anthranilate synthase). The tree was unrooted and inferred using IQ-Tree LG+C20+G model on an alignment of 263 taxa and 268 sites. UFBOOT (left) and SH-aLRT (right) support values are shown along the branches. Bacteria, Archaea and Woesearchaeota are colored in black, cyan and green, respectively. Woesearchaeota sequences are selected from 47 MAGs used in the inference of proteome content change history. Woesearchaeota subgroup J homologues are annotated in the figure. Scale bar shows the number of average substitutions per site. Source data are provided as a Source Data file and raw data are also available via figshare (See Data availability for more details).

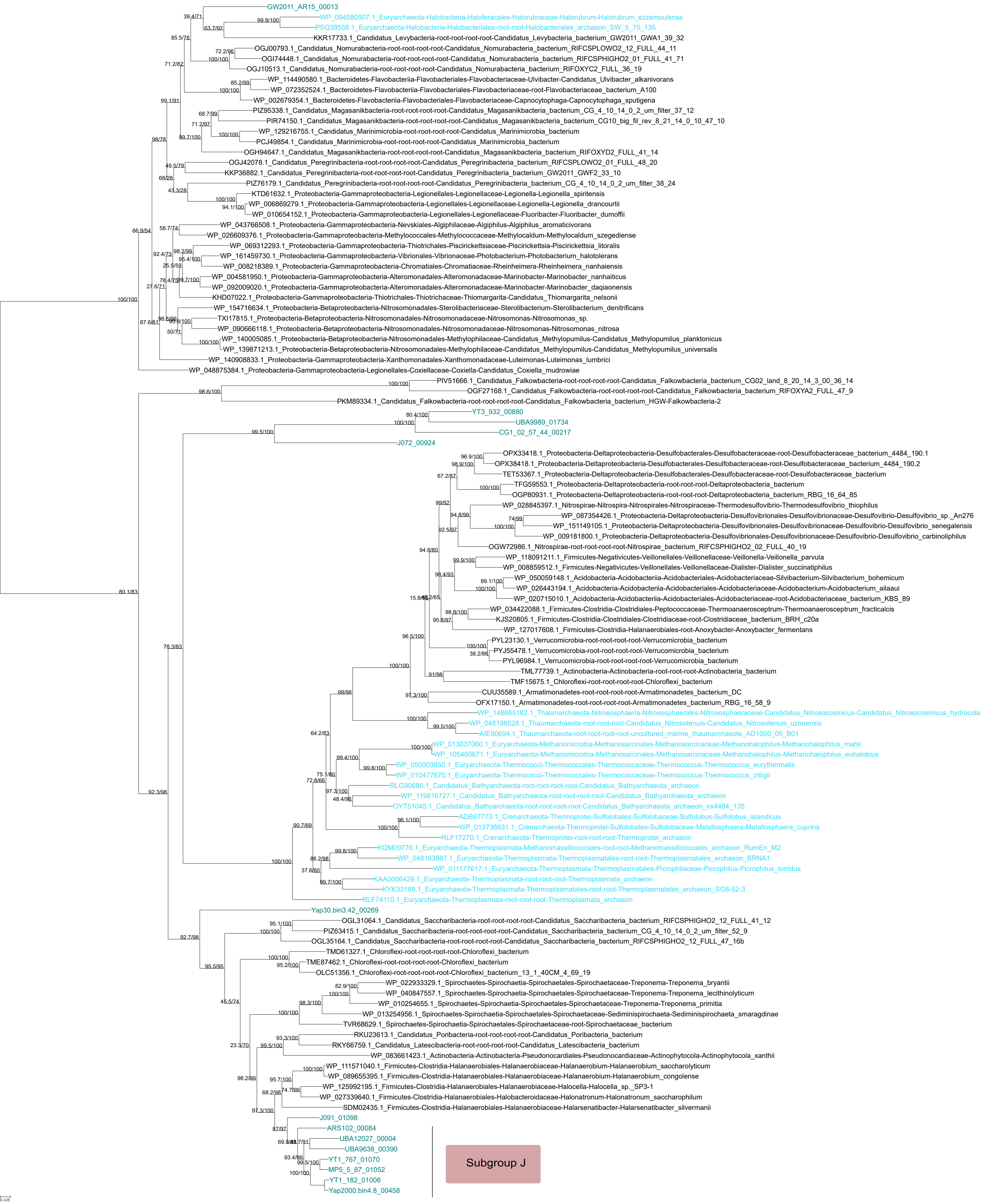

Supplementary Fig. 35: Phylogenetic analysis of *purB* gene (adenylosuccinate lyase). The tree was unrooted and inferred using IQ-Tree LG+C20+G model on an alignment of 115 taxa and 571 sites. UFBOOT (left) and SH-aLRT (right) support values are shown along the branches. Bacteria, Archaea and Woesearchaeota are colored in black, cyan and green, respectively. Woesearchaeota sequences are selected from 47 MAGs used in the inference of proteome content change history. Scale bar shows the number of average substitutions per site. Woesearchaeota subgroup J homologues are annotated in the figure. Source data are provided as a Source Data file and raw data are also available via figshare (See Data availability for more details).

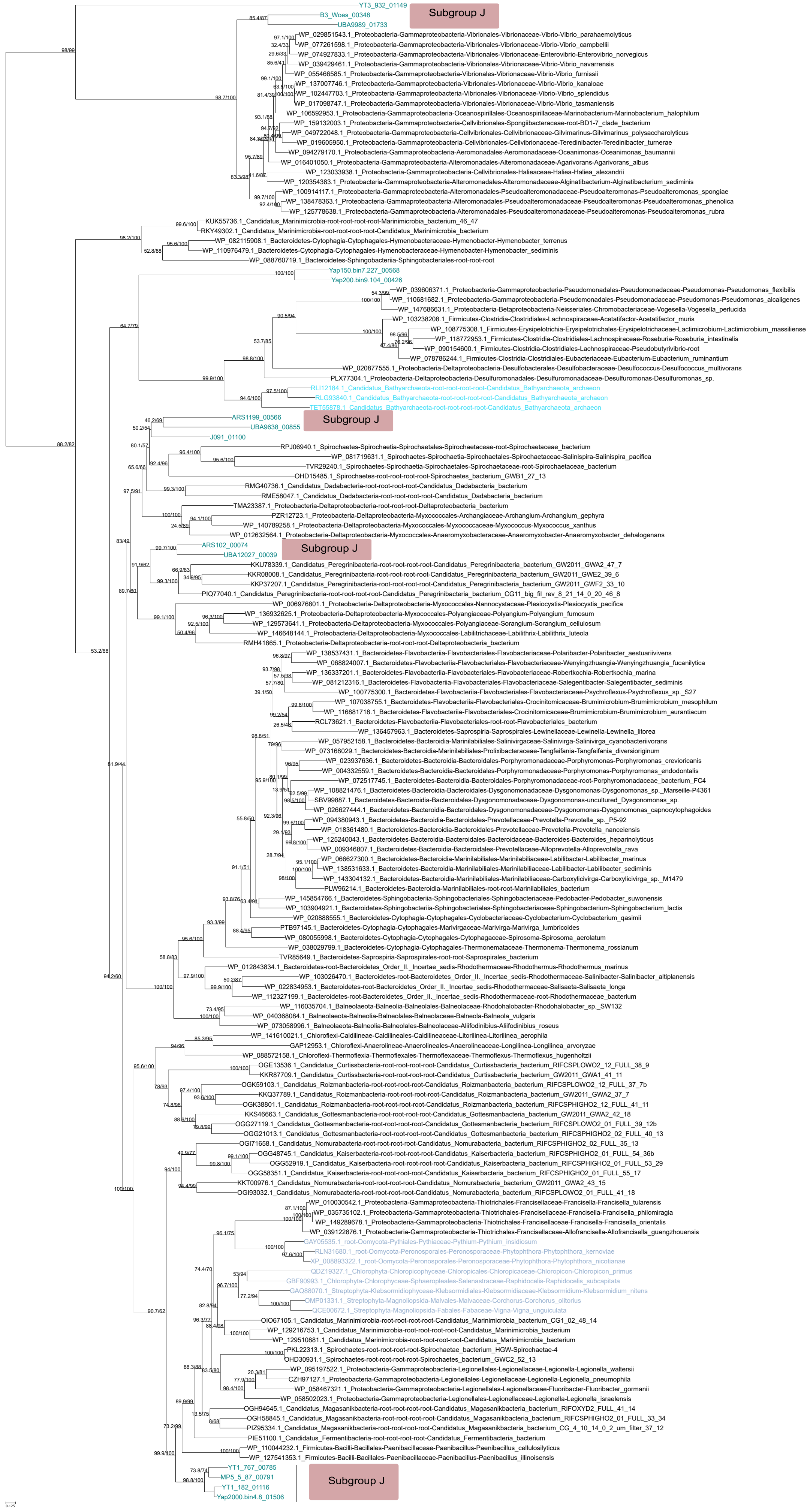

Supplementary Fig. 36: Phylogenetic analysis of *purC* gene (SAICAR synthetase). The tree was unrooted and inferred using IQ-Tree LG+C20+G model on an alignment of 153 taxa and 773 sites. UFBOOT (left) and SH-aLRT (right) support values are shown along the branches. Bacteria, Archaea, Woesearchaeota and Eukaryotes are colored in black, cyan, green and grey, respectively. Woesearchaeota sequences are selected from 47 MAGs used in the inference of proteome content change history. Scale bar shows the number of average substitutions per site. Woesearchaeota subgroup J homologues are annotated in the figure. Source data are provided as a Source Data file and raw data are also available via figshare (See Data availability for more details).

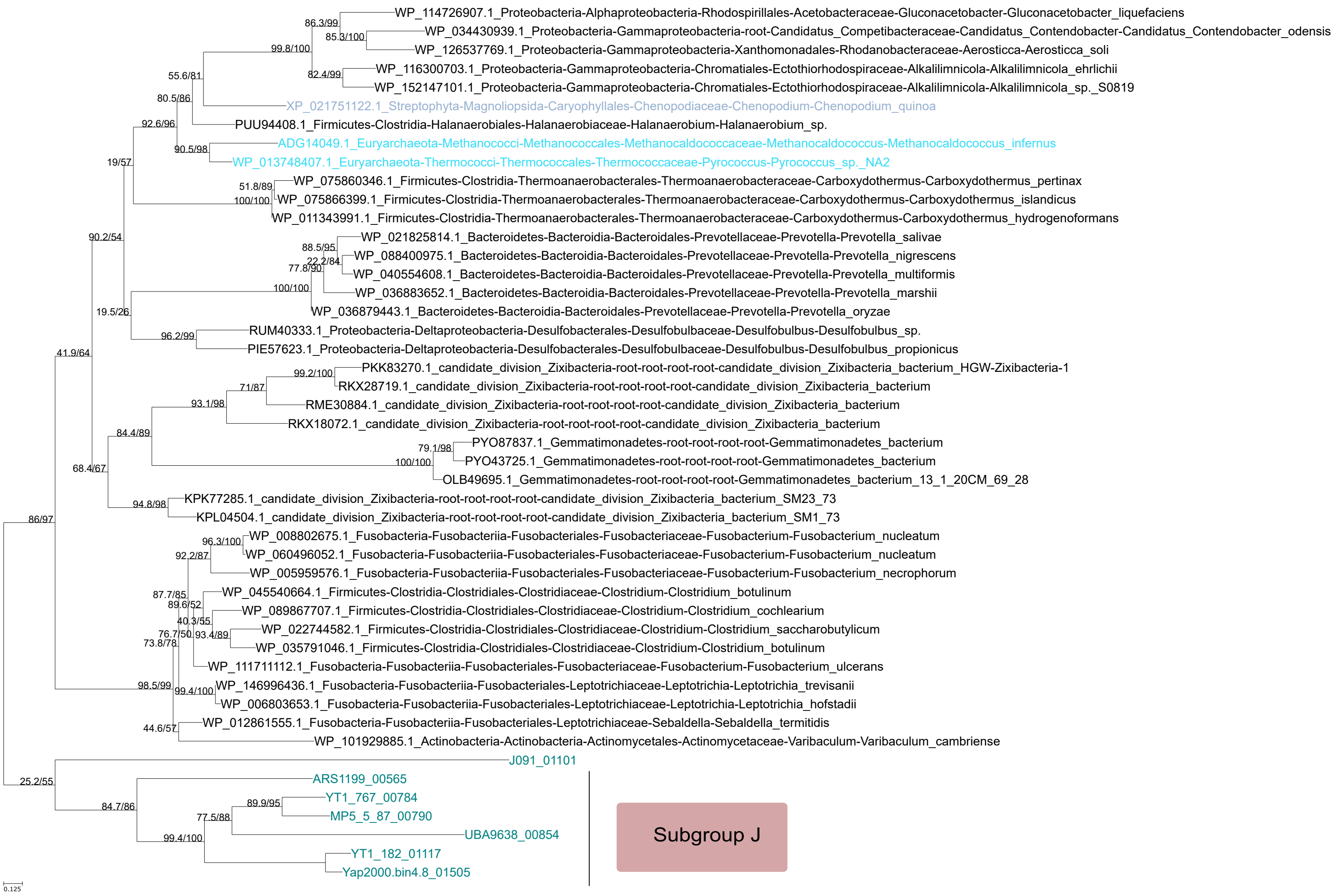

Supplementary Fig. 37: Phylogenetic analysis of *purE* gene (AIR carboxylase). The tree was unrooted and inferred using IQ-Tree LG+C20+G model on an alignment of 47 taxa and 509 sites. UFBOOT (left) and SH-aLRT (right) support values are shown along the branches. Bacteria, Archaea, Woesearchaeota and Eukaryotes are colored in black, cyan, green and grey, respectively. Woesearchaeota sequences are selected from 47 MAGs used in the inference of proteome content change history. Woesearchaeota subgroup J homologues are annotated in the figure. Scale bar shows the number of average substitutions per site. Source data are provided as a Source Data file and raw data are also available via figshare (See Data availability for more details).

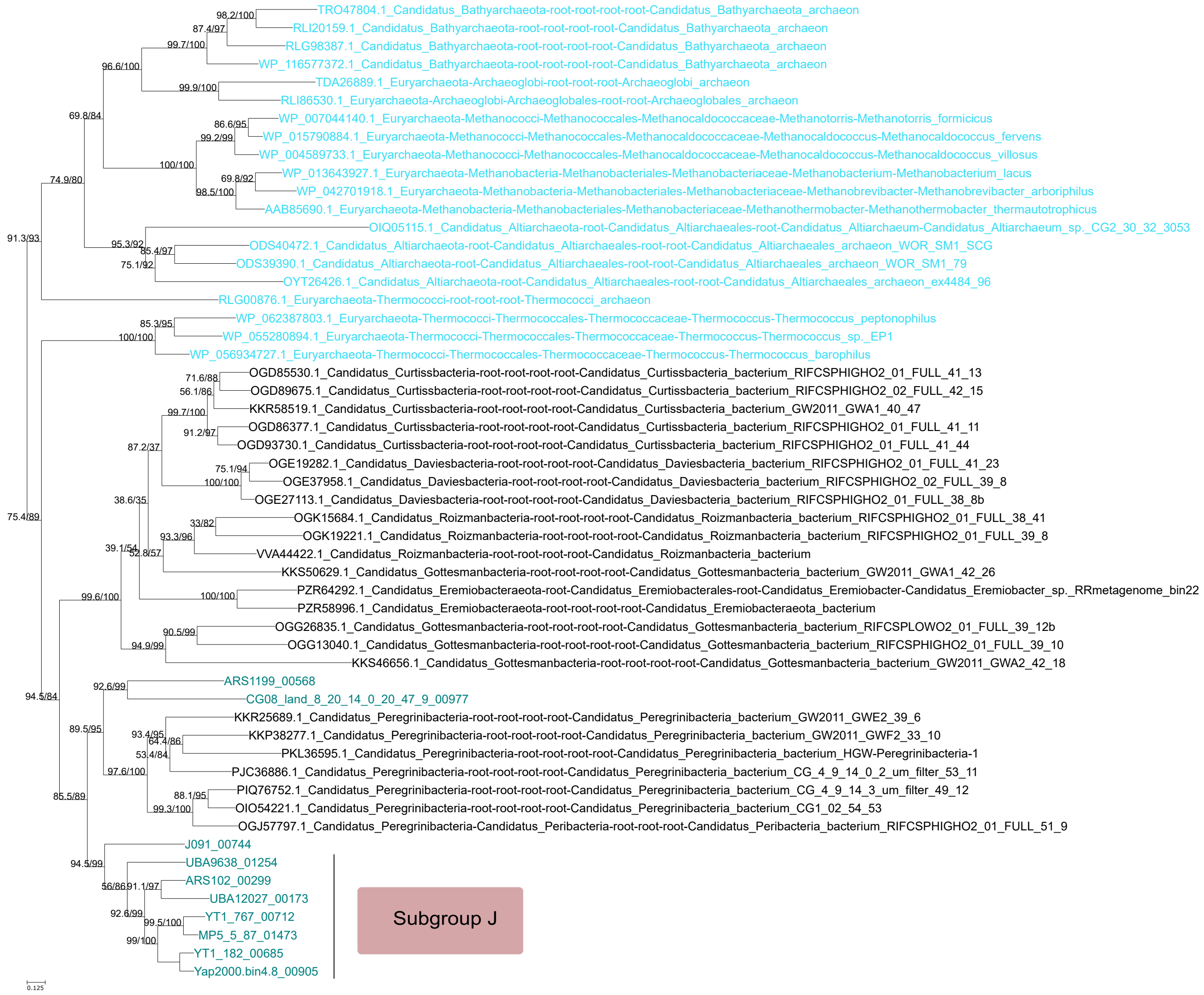

Supplementary Fig. 38: Phylogenetic analysis of *purP* gene (5-formaminoimidazole-4-carboxamide-1-(beta)-D-ribofuranosyl 5'-monophosphate synthetase). An unrooted phylogenetic tree was inferred using IQ-Tree LG+C20+G model on an alignment of 59 taxa and 544 sites. UFBOOT (left) and SH-aLRT (right) support values are shown along the branches. Bacteria, Archaea and Woesearchaeota are colored in black, cyan and green, respectively. Woesearchaeota sequences are selected from 47 MAGs used in the inference of proteome content change history. Scale bar shows the number of average substitutions per site. Woesearchaeota subgroup J homologues are annotated in the figure. Source data are provided as a Source Data file and raw data are also available via figshare (See Data availability for more details).

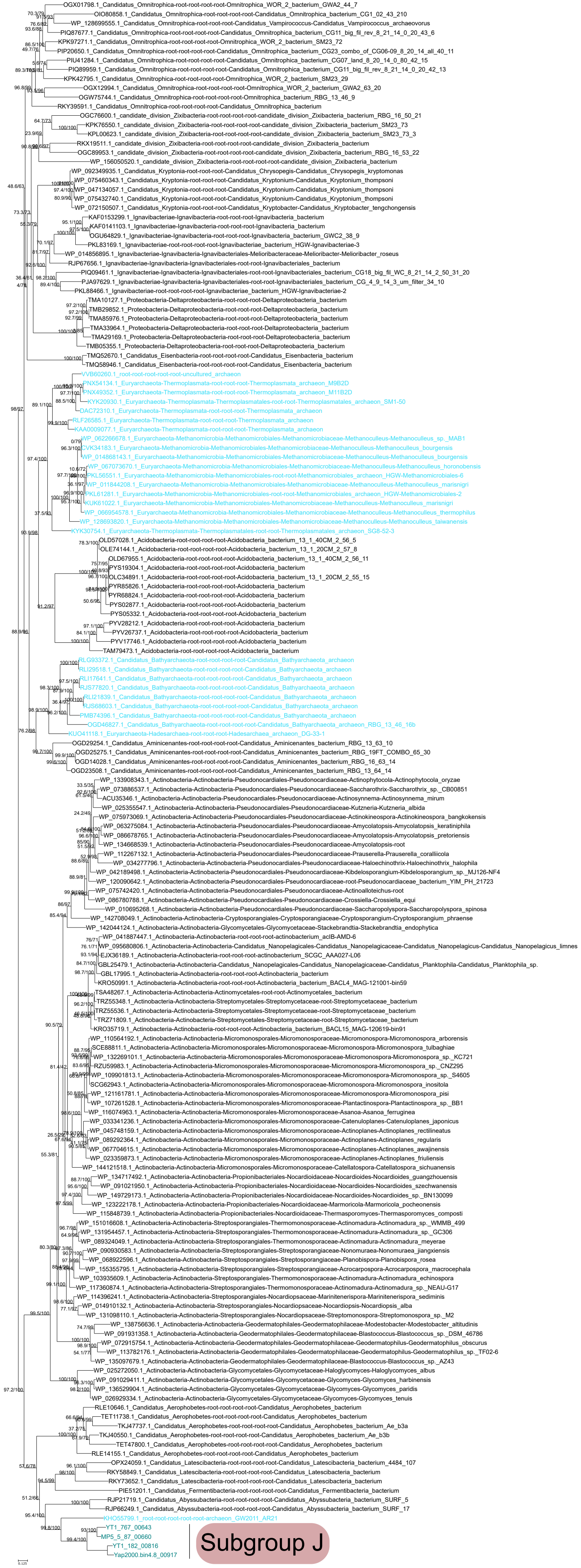

Supplementary Fig. 39: Phylogenetic analysis of *pfk* gene (phosphofrutokinase). The tree was unrooted and inferred using IQ-Tree LG+G+F+C20 model on an alignment of 169 taxa and 351 sites. UFBOOT (left) and SH-aLRT (right) support values are shown along the branches. Bacteria, Archaea and Woesearchaeota are colored in black, cyan and green, respectively. Woesearchaeota sequences are selected from the subgroup J MAGs. Scale bar shows the number of average substitutions per site. Source data are provided as a Source Data file and raw data are also available via figshare (See Data availability for more details).

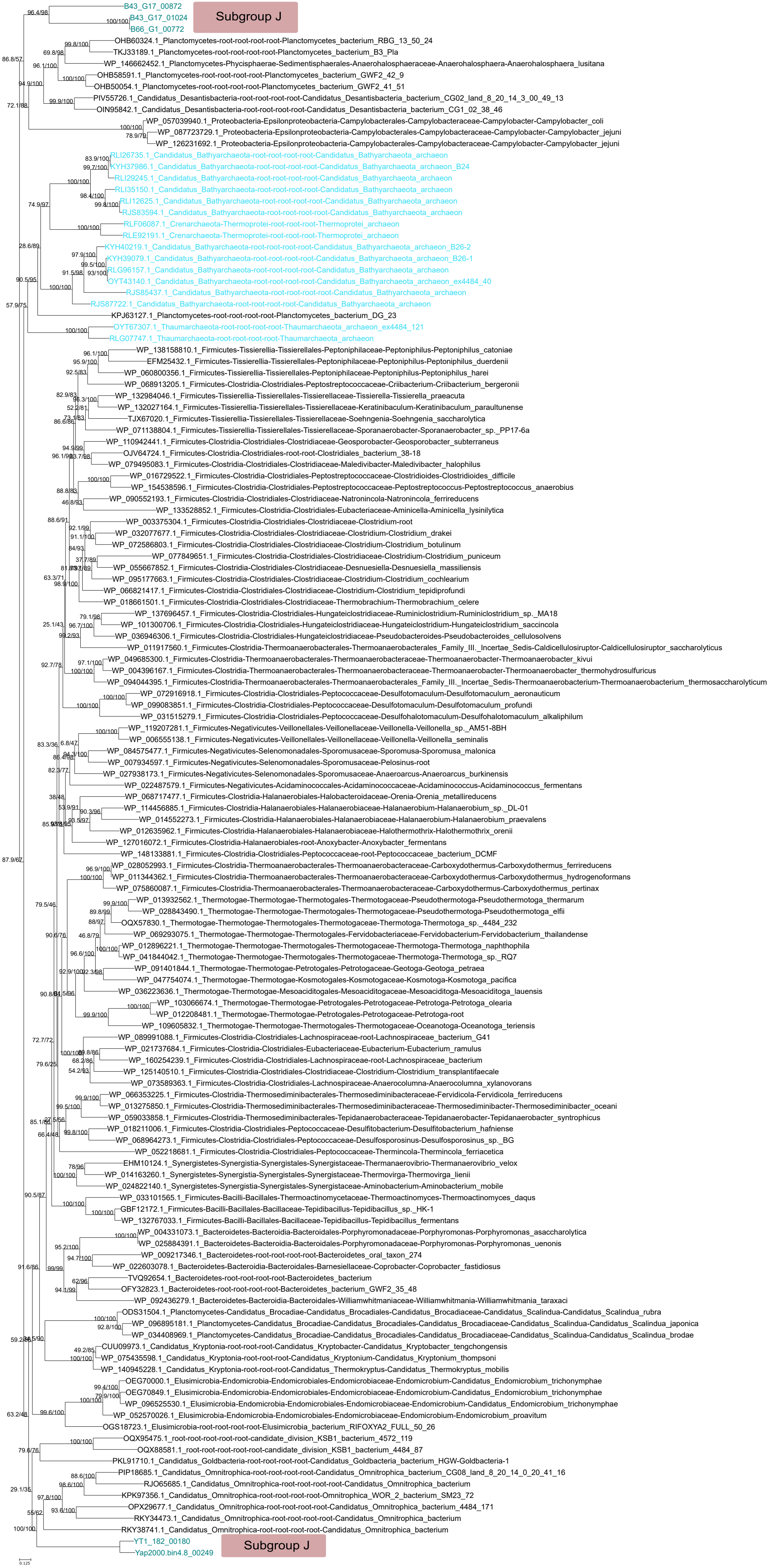

Supplementary Fig. 40: Phylogenetic analysis of *ackA* gene (Acetate kinase). The tree was unrooted and inferred using IQ-Tree LG+G+F+C20 model on an alignment of 136 taxa and 400 sites. UFBOOT (left) and SH-aLRT (right) support values are shown along the branches. Bacteria, Archaea and Woesearchaeota are colored in black, cyan and green, respectively. Woesearchaeota sequences are selected from the subgroup J MAGs. Scale bar shows the number of average substitutions per site. Source data are provided as a Source Data file and raw data are also available via figshare (See Data availability for more details).

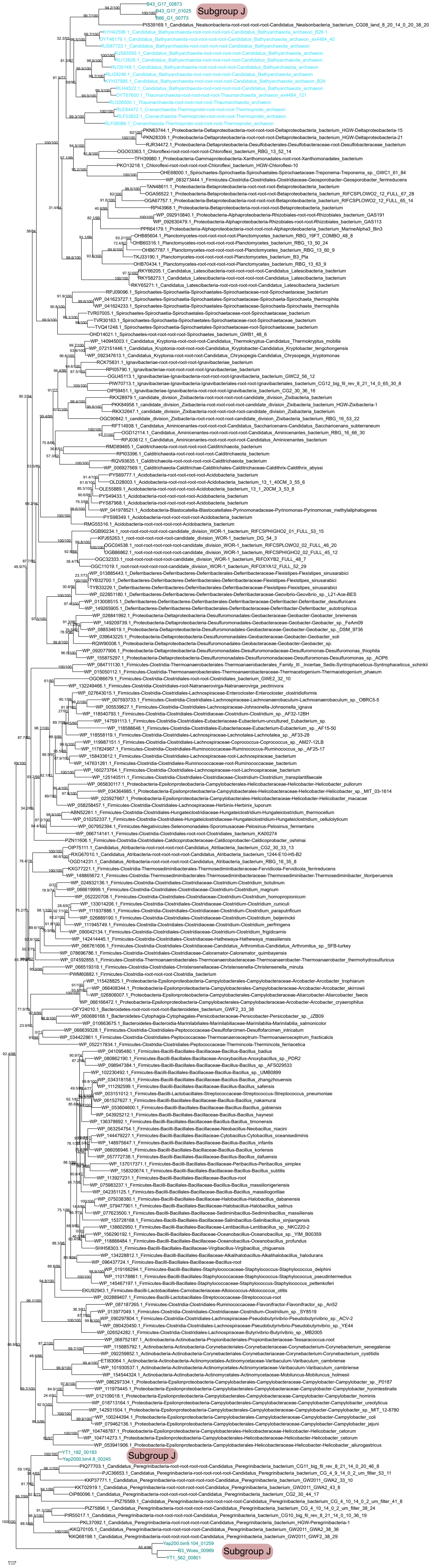

Supplementary Fig. 41: Phylogenetic analysis of *pta* gene (Phosphate acetyltransferase). The tree was unrooted and inferred using IQ-Tree LG+C50 +F+I+G model on an alignment of 222 taxa and 557 sites. UFBOOT (left) and SH-aLRT (right) support values are shown along the branches. Bacteria, Archaea and Woesearchaeota are colored in black, cyan and green, respectively. Scale bar shows the number of average substitutions per site. Woesearchaeota sequences are selected from the subgroup J MAGs. Source data are provided as a Source Data file and raw data are also available via figshare (See Data availability for more details).

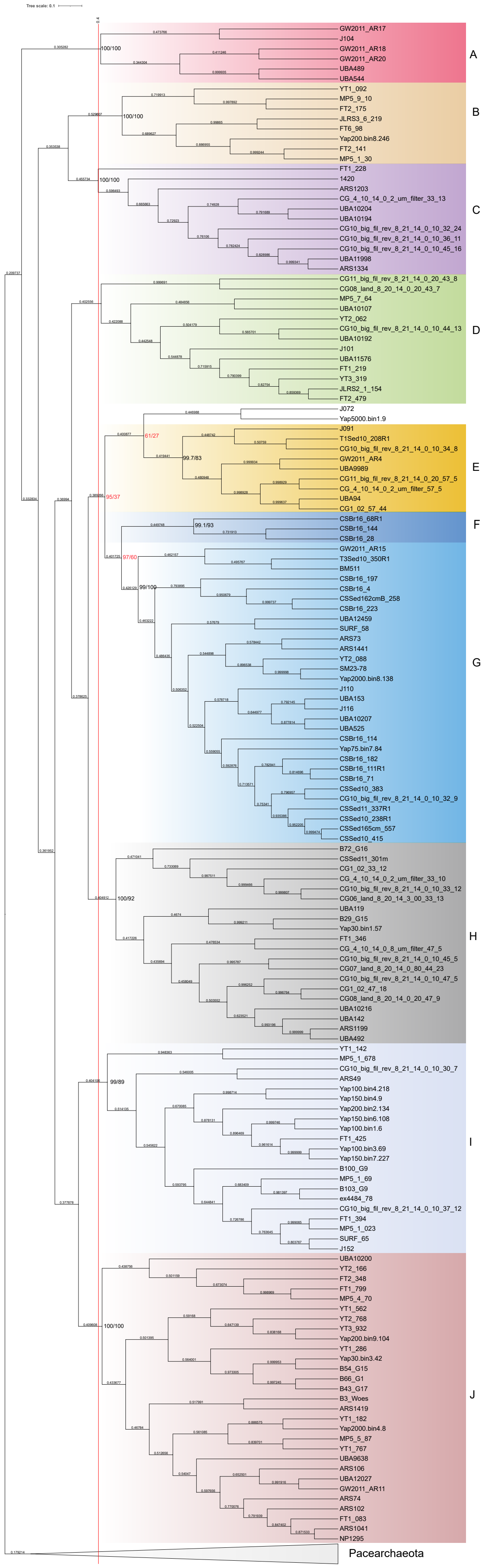

Supplementary Fig. 42: Ultrametric tree of the phylogenetic analysis of 152 woesearchaeotal MAGs using IQ-Tree (v 2.0.7) LG+F+R+C60 model on the concatenated 50% top-ranked marker proteins. Red line denotes a branch length of 0.4 of the ultrametric tree. The numbers at the middle of the branch are the relative evolutionary divergence value calculated from the original tree (the tree shown in Fig. 2). UFBOOT (left) and SH-aLRT (right) support values are shown behind the node for subgroup division. Support values colored in red indicates the reliability of the clade is not strongly supported and therefore subgroup was assigned at the next node whose UFBOOT >= 95% and SH-aLRT >= 80%.

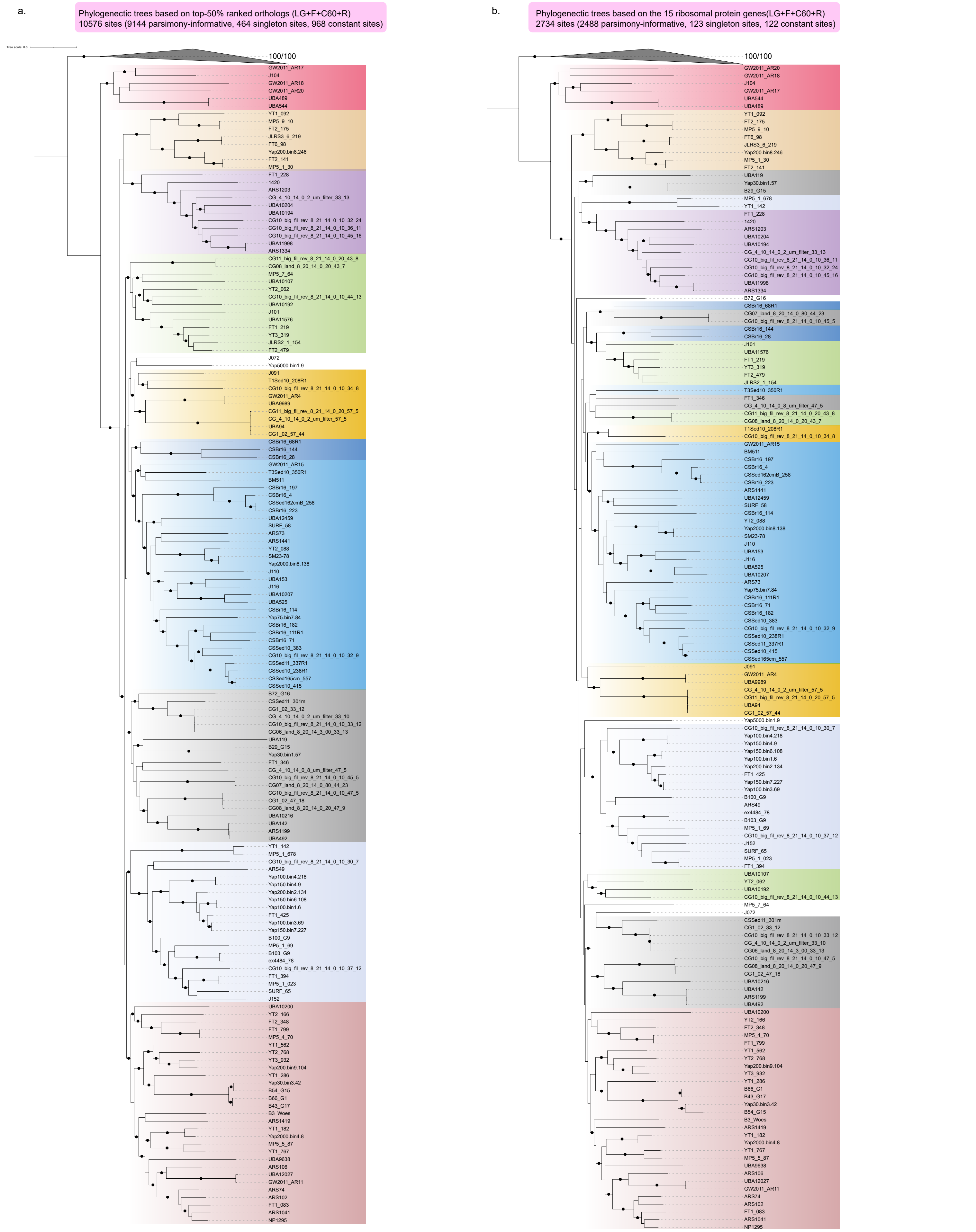

Supplementary Fig. 43: Comparison of subgroup in the top-50% ranked orthologs trees and 15 ribosomal protein trees. a. Phylogenetic tree based on the top-50% ranked orthologs tree. **b**. Phylogenetic tree based on 15 ribosomal proteins. Genomes are colored according to the subgroup assigned using the top-50% ranked orthologs tree. Scale bar shows average substitution per site. Black dots indicate nodes with support value (UFBOOT >= 95% and SH-aLRT >= 80%). Source data are provided as a Source Data file and raw data are also available via figshare (See Data availability for more details).

References:

1. Liu, X. *et al.* Insights into the ecology, evolution, and metabolism of the widespread Woesearchaeotal lineages. *Microbiome* **6**, 102 (2018).
2. Karst, S. M. *et al.* Retrieval of a million high-quality, full-length microbial 16S and 18S rRNA gene sequences without primer bias. *Nature Biotechnology* **36**, 190–195 (2018).
