# Supplementary material for: Comparative genomic analysis reveals metabolic flexibility of Woesearchaeota
Source: Nat Commun. 2021 Sep 6;12:5281. doi: 10.1038/s41467-021-25565-9 (PMC8421398; doi:10.1038/s41467-021-25565-9)
Supplement: Supplementary file 3 — Description of Additional Supplementary Files [file 41467_2021_25565_MOESM3_ESM.pdf]

Description of Additional Supplementary Files

File Name: Supplementary Data 1
Description: Metagenome-assembled genomes (MAGs) used in current study. All MAGs are medium-quality draft assemblies (completeness ≥50%; contamination ≤10%). Completeness and contamination were estimated with CheckM as described in Methods. "NA" in "Subgroup" column indicates that the term is not applicable for the genome.

File Name: Supplementary Data 2
Description: Metadata of 2163 16S rRNA gene libraries used for exploration of Woesearchaeota ecological pattern.

File Name: Supplementary Data 3
Description: Annotations of Woesearchaeota genomes. The genomes were ordered according to the phylogenetic tree shown in Fig. 2.

File Name: Supplementary Data 4
Description: Gene gain events predicted at the six nodes (node 92 to node 91, node 91 to node 87, node 87 to node 82, node 82 to node 73, node 82 to node 79, node 79 to 74). OG refers to orthologous groups obtained with Orthofinder as described in Methods. Name refers to preferred gene name used in eggNOG (v5.0) clusters and COG category was sourced from first cluster of orthologous groups [COG] hit reported by eggNOG-mapper (v2). Blank indicates that no COG category was assigned to the orthologous group.

File Name: Supplementary Data 5
Description: Assessment and evaluation of orthologs suitable for concatenation for phylogenetic analysis. The top 50% ranked orthologs included orthologs with (Rank A+B) < 75.

File Name: Supplementary Data 6
Description: Detailed annotations of selected genes from Woesearchaeota genomes (YT1_182 and Yap2000.bin4.8) and pathways of interest. Annotations include (if available) eggNOG, CDD, TIGRFAM, Pfam and SMART.
