# Supplementary material for: Comparative genomic analysis reveals metabolic flexibility of Woesearchaeota
Source: Nat Commun. 2021 Sep 6;12:5281. doi: 10.1038/s41467-021-25565-9 (PMC8421398; doi:10.1038/s41467-021-25565-9)
Supplement: Supplementary file 10 — Reporting Summary [file 41467_2021_25565_MOESM10_ESM.pdf]

nature research

Corresponding author(s): Meng Li

Last updated by author(s): Jul 13, 2021

# Reporting Summary

Nature Research wishes to improve the reproducibility of the work that we publish. This form provides structure for consistency and transparency in reporting. For further information on Nature Research policies, see our Editorial Policies and the Editorial Policy Checklist.

## Statistics

For all statistical analyses, confirm that the following items are present in the figure legend, table legend, main text, or Methods section.

| n/a | Confirmed | |
|---|---|---|
| ☐ | ☒ | The exact sample size (*n*) for each experimental group/condition, given as a discrete number and unit of measurement |
| ☒ | ☐ | A statement on whether measurements were taken from distinct samples or whether the same sample was measured repeatedly |
| ☐ | ☒ | The statistical test(s) used AND whether they are one- or two-sided<br>*Only common tests should be described solely by name; describe more complex techniques in the Methods section.* |
| ☒ | ☐ | A description of all covariates tested |
| ☒ | ☐ | A description of any assumptions or corrections, such as tests of normality and adjustment for multiple comparisons |
| ☐ | ☒ | A full description of the statistical parameters including central tendency (e.g. means) or other basic estimates (e.g. regression coefficient) AND variation (e.g. standard deviation) or associated estimates of uncertainty (e.g. confidence intervals) |
| ☐ | ☒ | For null hypothesis testing, the test statistic (e.g. *F*, *t*, *r*) with confidence intervals, effect sizes, degrees of freedom and *P* value noted<br>*Give P values as exact values whenever suitable.* |
| ☒ | ☐ | For Bayesian analysis, information on the choice of priors and Markov chain Monte Carlo settings |
| ☒ | ☐ | For hierarchical and complex designs, identification of the appropriate level for tests and full reporting of outcomes |
| ☐ | ☒ | Estimates of effect sizes (e.g. Cohen's *d*, Pearson's *r*), indicating how they were calculated |

*Our web collection on statistics for biologists contains articles on many of the points above.*

## Software and code

Policy information about availability of computer code

| | |
|---|---|
| Data collection | No software was used for data collection |
| Data analysis | Custom python scripts (to analyze tree files) and R codes (to perform ecological analysis) have been deposited at figshare (https://doi.org/10.6084/m9.figshare.14459535). Open source software used in analysis is referenced in Methods: R (v3.6.0), python (v3.7.0), Rtsne (v0.15 ), vegan (v2.5-7), Sickle (v1.33), VSEARCH (v2.13.3), uclust (v1.2.22q), QIIME (v2018.11), metaWRAP (v1.2.4), MEGAHIT (v1.1.3), IDBA-UD (v1.1.1), Sickle (v1.33), MetaBAT2 (v2.12.1), Das-Tool (v1.0), CheckM (v1.0.12), prodigal (v2.6.3), prokka (v1.13) , Barrnap (v0.9), Clipkit (v0.1), InterProScan (v5.38-76.0, client version), eggNOG-mapper (v2) , hmmsearch (v3.1b2), DIAMOND (v0.9.24), OMA standalone (v2.4.1), BMGE (v1.12), MAFFT (v7.471), GraftM (v0.12.2), FastTree (v2.1.10), IQ-Tree (v1.6.8; v2.0.7), trimAL (v1.4.rev15), Orthofinder (v2.2.6), ClipKIT (v0.1), KofamScan (v1.3.0), ALEml_undated (v1.0), ETE Toolkit (v3.1.2), Cd-hit (v4.8.1), DNA Features Viewer (v3.0.3), Trimmomatic (v0.38) logomaker (v0.8) and iTOL v4.<br><br>Public database used in this study included SILVA SSU 132 database (https://www.arb-silva.de/documentation/release-132), Greengenes (v13_8) [http://greengenes.microbio.me/greengenes_release/gg_13_8_otus/], CDD (v3.17) [ftp://ftp.ncbi.nih.gov/pub/mmdb/cdd/], Pfam (v 32.0) [ftp://ftp.ebi.ac.uk/pub/databases/Pfam/releases/], SMART (7.1) [https://ftp.ebi.ac.uk/pub/software/unix/iprscan/5/5.38-76.0/], TIGRFAM (v 15.0) [ftp://ftp.jcvi.org/pub/data/TIGRFAMs], KEGG KOfam (ver. 2020-06-07) [https://www.genome.jp/tools/kofamkoala/], Carbohydrate-active enzymes (CAZymes) database downloaded from dbCAN2 in July 2019 [http://bcb.unl.edu/dbCAN2/download/], MEROPS (v 12.0) [ftp://ftp.ebi.ac.uk/pub/databases/merops/old_releases/merops120], Transporter Classification Database downloaded in November 2020 [http://www.tcdb.org/download.php], eggNOG (v 5.0) [http://eggnog5.embl.de/download/eggnog_5.0/], arCOG [http://eggnog5.embl.de/download/eggnog_5.0/per_tax_level/2157/], HydDB [https://services.birc.au.dk/hyddb/], NCBI-nr database downloaded in March 2020 [ftp://ftp.ncbi.nlm.nih.gov/blast/db/] and the archaeal and bacterial backbone datasets in Dombrowski et al. [https://doi.org/10.5281/zenodo.3672835]. Source data are provided with this paper. |

For manuscripts utilizing custom algorithms or software that are central to the research but not yet described in published literature, software must be made available to editors and reviewers. We strongly encourage code deposition in a community repository (e.g. GitHub). See the Nature Research guidelines for submitting code & software for further information.

# Data

Policy information about availability of data

All manuscripts must include a data availability statement. This statement should provide the following information, where applicable:

- Accession codes, unique identifiers, or web links for publicly available datasets
- A list of figures that have associated raw data
- A description of any restrictions on data availability

The Woesearchaeota MAGs have been deposited in eLMSG (an eLibrary of Microbial Systematics and Genomics, https://www.biosino.org/elmsg/index) and are also available from the NCBI under the BioProject identifier PRJNA746083. The accession number for the MAGs are available in Supplementary Data 1. DNA sequencing data are deposited in the NCBI SRA under the BioProject identifier PRJNA746083 and PRJNA680430.

Public database used in this study included SILVA SSU 132 database (https://www.arb-silva.de/documentation/release-132), Greengenes (v13_8) [http://greengenes.microbio.me/greengenes_release/gg_13_8_otus/], CDD (v3.17) [ftp://ftp.ncbi.nih.gov/pub/mmdb/cdd/], Pfam (v 32.0) [ftp://ftp.ebi.ac.uk/pub/databases/Pfam/releases/], SMART (7.1) [https://ftp.ebi.ac.uk/pub/software/unix/iprscan/5/5.38-76.0/], TIGRFAM (v 15.0) [ftp://ftp.jcvi.org/pub/data/TIGRFAMs], KEGG KOfam (ver. 2020-06-07) [https://www.genome.jp/tools/kofamkoala/], Carbohydrate-active enzymes (CAZymes) database downloaded from dbCAN2 in July 2019 [http://bcb.unl.edu/dbCAN2/download/], MEROPS (v 12.0) [ftp://ftp.ebi.ac.uk/pub/databases/merops/old_releases/merops120], Transporter Classification Database downloaded in November 2020 [http://www.tcdb.org/download.php], eggNOG (v 5.0) [http://eggnog5.embl.de/download/eggnog_5.0/], arCOG [http://eggnog5.embl.de/download/eggnog_5.0/per_tax_level/2157/], HydDB [https://services.birc.au.dk/hyddb/], NCBI-nr database downloaded in March 2020 [ftp://ftp.ncbi.nlm.nih.gov/blast/db/] and the archaeal and bacterial backbone datasets in Dombrowski et al. [https://doi.org/10.5281/zenodo.3672835]. Source data are provided with this paper.

# Field-specific reporting

Please select the one below that is the best fit for your research. If you are not sure, read the appropriate sections before making your selection.

☐ Life sciences ☐ Behavioural & social sciences ☒ Ecological, evolutionary & environmental sciences

For a reference copy of the document with all sections, see nature.com/documents/nr-reporting-summary-flat.pdf

# Ecological, evolutionary & environmental sciences study design

All studies must disclose on these points even when the disclosure is negative.

| | |
|---|---|
| Study description | We investigated Woesearchaeota distribution patterns and the factors that influence their community. Further we divided them into ten phylogenetically distinct subgroups, reevaluated their metabolic potentials and reconstructed their evolutionary history using gene tree-species tree reconciliation techniques. |
| Research sample | Analysis involved 2163 16S rRNA gene amplicon sequencing datasets (https://earthmicrobiome.org/protocols-and-standards/metadata-guide/) and 152 Woesearchaeota genomes, including 103 genomes downloaded at November 8, 2019 from NCBI (www.ncbi.nlm.nih.gov), IMG (https://img.jgi.doe.gov/) and GTDB (release 89; https://data.ace.uq.edu.au/public/gtdb/data/releases/), and 49 new ones reconstructed in this study. The 49 genome sequences were recovered from marine water (Yap metagenomes, collected at Yap trench region by CTD SBE911plus during the 37th Dayang cruise in 2016), sediments of mangrove (FT metagenomes, taken from Futian Nature Reserve in April 17, 2017; MP5 metagenomes, obtained from Mai Po Nature Reserve in September 12, 2014), sediments of seagrass bed (YT metagenomes, obtained from the Rongcheng Nation Swan Nature Reserve in November 15, 2018) and sediments of JiuLong River estuary (JLR metagenomes, taken from Jiulong River estuary using a grabber during a cruise in November 28, 2018. |
| Sampling strategy | 16S rRNA gene sequencing libraries were selected if they have greater than 3 Woesearchaeota sequences after rarefaction. These libraries were selected from earth microbiome project dataset. The selection should hold enough information to explore Woesearchaeota distribution patterns. Metagenome-assembled genomes of Woesearchaeota were downloaded from public database and assembled from metagenomic sequence libraries. This should make a comprehensive dataset of Woesearchaeota genomes. These genomes were further screened for acceptable quality using standard estimates (CheckM) of completeness (≥50%) and contamination (≤10%) to interpret the genome content. |
| Data collection | Information of the 16S rRNA gene amplicon sequencing datasets were downloaded from EMP website (https://earthmicrobiome.org/protocols-and-standards/metadata-guide/) and NCBI (www.ncbi.nlm.nih.gov/sra) by Mingwei Cai. The public Woesearchaeota genomes were downloaded from the NCBI (www.ncbi.nlm.nih.gov), IMG (https://img.jgi.doe.gov/), and GTDB database (release 89; https://data.ace.uq.edu.au/public/gtdb/data/releases/) by Wen-Cong Huang.<br><br>For each YT sample, DNA was extracted from 10 g sediment using PowerSoil DNA Isolation kit (QIAGEN, Germany), according to the manufacturer's protocol. Following extraction, nucleic acids were sequenced using Illumina HiSeq2500 (Illumina, USA) PE150 by Novogene (Nanjing, China). Yang Liu, Wen-Cong Huang, Shiling Zheng and Fanghua Liu (Yantai Institute of Coastal Zone Research, Chinese Academy of Sciences) were present during the data collection.<br>For MP samples, metagenomic sequencing data were generated using Illumina HiSeq2500 (Illumina, USA) PE150 by Novogene (Tianjin, China). Zhichao Zhou and Ji-Dong Gu (The University of Hong Kong) were present during the data collection.<br>For FT samples, nucleic acids were sequenced using Illumina HiSeq2000 (Illumina, USA) PE150 by Novogene (Tianjin, China). Yang Liu, Wen-Cong Huang and colleagues from Shenzhen University were present during the data collection.<br>For the seawater samples of Yap trench, nucleic acids were sequenced using HiSeq X Ten (Illumina, USA) PE150. Wei Xu and Zhuhua Luo were present during the data collection. |

For the JLR samples, DNA was extracted with PowerMax soil kit (Qiagen) as per manufacturer's instructions and sequenced using HiSeq 2000 PE150 (Illumina, USA). Ru Wan and Shuh-Ji Kao were present during the data collection.

Timing and spatial scale

All public data analyzed were collected by November 8, 2019.
YT samples were obtained from the Rongcheng Nation Swan Nature Reserve (Rongcheng, China) in 15th November 2018. The sediment cores were collected using columnar samplers at depth intervals of 0–2, 21–26, and 36–41 cm at a seagrass meadow and a non-seagrass–covered site nearby.
MP5 samples were obtained from Mai Po Nature Reserve (Hong Kong, China) in 12th September 2014. Three subsurface sediments samples were collected from a site covering with mangrove forest at depth intervals of 0-2, 10-15 and 20-25 cm. Two subsurface sediment samples were taken at an intertidal mudflat with depths of 0-5 and 13-16 cm.
FT samples were taken from Futian Nature Reserve (Shenzhen, Guangdong, China) in 17th April 2017. Sediment samples were collected as described for YT samples at depth intervals of 0-2, 6-8, 12-14, 20-22, and 28-30 cm.
The seawater samples of Yap metagenomes were collected at Yap trench region by CTD SBE911plus (Sea-Bird Electronics, USA) during the 37th Dayang cruise during 4th June – 12th July 2016.
The surface sediment of JLR samples were collected at Jiulong River esturay using a grabber during a cruise in 28th November 2018.
These sampling and data collection should be enough for a discovery project.

Data exclusions

Woesearchaeota genomes with an estimated completeness lower than 50% or contamination greater than 10% were excluded from the study. These genomes are excluded as they represent low quality genomes and may negatively impact the quality of data interpretation and inferred phylogeny.

Reproducibility

This project was based on bioinformatics analyses and all software versions and bioinformatics analysis are documented in details in the manuscript to reproduce the results.

Randomization

All 16S rRNA gene dataset and metagenome-assembled genomes of Woesearchaeota were included in the analysis to investigate their distribution pattern and metabolic capacity. Therefore, randomization is not relevant to this study.

Blinding

As a discovery project, this study investigated distribution pattern and metabolic capacity of different Woesearchaeota. All collected data are analyzed, so this is not relevant to the study.

Did the study involve field work? ☐ Yes ☒ No

# Reporting for specific materials, systems and methods

We require information from authors about some types of materials, experimental systems and methods used in many studies. Here, indicate whether each material, system or method listed is relevant to your study. If you are not sure if a list item applies to your research, read the appropriate section before selecting a response.

## Materials & experimental systems

| n/a | Involved in the study |
|---|---|
| ☒ | ☐ Antibodies |
| ☒ | ☐ Eukaryotic cell lines |
| ☒ | ☐ Palaeontology and archaeology |
| ☒ | ☐ Animals and other organisms |
| ☒ | ☐ Human research participants |
| ☒ | ☐ Clinical data |
| ☒ | ☐ Dual use research of concern |

## Methods

| n/a | Involved in the study |
|---|---|
| ☒ | ☐ ChIP-seq |
| ☒ | ☐ Flow cytometry |
| ☒ | ☐ MRI-based neuroimaging |
